# Association between lipoproteins and telomere length in US adults: data from the NHANES 1999–2002

**DOI:** 10.1186/s12944-019-1030-7

**Published:** 2019-04-01

**Authors:** Yun-Fen Chen, Kai-Wen Zhou, Gui-zhen Yang, Chi Chen

**Affiliations:** 1 Department of Nephropathy of the people’s hospital of Guizhou province, 49 # Zhongshan East road, Guiyang, 550001 Guizhou China; 2 School of Medicine and Nursing of Dezhou College, 566# Decheng District University West Road, Dezhou City, 553433 Shandong China; 3 Department of Immunology and Microbiology and Statistical Epidemiology, Guizhou University of Traditional Chinese Medicine, 84# ShiDong Road, Guiyang, 550001 Guizhou China; 4 Department of Immunology and Microbiology, Guiyang College of Traditional Chinese Medicine, 84# ShiDong Road, Guiyang, 550001 Guizhou China

**Keywords:** Lipoprotein, Telomere length, Association, Nonlinearity, NHANES database

## Abstract

**Background:**

Evidence regarding the correlation between lipoproteins and telomere length in US adults is limited. We aimed to investigate whether lipoproteins was associated with telomere length using US National Health and Nutrition Examination Survey (NHANES) database.

**Methods:**

A total of 6468 selected participants were identified in the NHANES Data Base (1999–2002). The independent and dependent variables were lipoproteins and telomere length, respectively. The covariates included demographic data, dietary data, physical examination data, and comorbidities.

**Results:**

In fully-adjusted model, we found that 0.1 differences of telomere length were positively associated with HDL-C [0.19 (95% CI 0.07, 0.31)], while the associations between LDL-C [0.19 (95% CI -0.27, 0.65)], TG [− 1.00 (95% CI -2.09, 0.07) and telomere length were not detected. By nonlinearity test, only the relationship between HDL-C and telomere length was nonlinear. The inflection point we got was 1.25. On the left side of the inflection point (telomere length ≤ 1.25), a difference in 0.1 of telomere length was associated with 0.50 difference in HDL-C.

**Conclusion:**

After adjusting for demographic data, dietary data, physical examination data, and comorbidities, telomere length is not associated with LDL-C and TG, but is positively associated with HDL-C when telomere length is less than 1.25.

**Electronic supplementary material:**

The online version of this article (10.1186/s12944-019-1030-7) contains supplementary material, which is available to authorized users.

## Background

Epidemiological evidence has shown lipoproteins are correlated with the risk of cardiovascular disease (CVD) [1–3]. Besides, a large body of evidence also suggests that leukocyte telomere length is an independent risk factor of CVD, irrespective of adjustment for conventional risk factors [4]. Under the circumstances, Rehkopf, D. H, et al [5] utilizes the US National Health and Nutrition Examination Survey (NHANES) database to investigate whether telomere length is independent from current known risk factors (including lipoproteins) for cardiovascular disease. Their findings have suggested that high density lipoprotein cholesterol (HDL-C) and triglycerides (TG) is related to telomere length. Yet, the focus of their study is not only on lipoproteins, but also includes 14 other risk factors of CVD. Their data analysis is therefore not in-depth enough. In the present study, we used the same NHANES data to re-evaluate the association for lipoproteins with telomere length.

## Materials and methods

### Data source

NHANES, an ongoing repeated cross-sectional study conducted by the US National Center for Health Statistics (NCHS), is a nationwide database that contains information about the health and nutritional status of adults and children in the United States. The NHANES has collected data since 1999 and includes unique information about interviews and physical examinations. The data from NHANES official website (http://www.cdc.gov/nchs/nhanes/nhanes_questionnairees.htm) were analysed and presented. NHANES protocol was approved by NCHS Research Ethics Review Board, and informed consent were obtained from all participants. The NHANES database consists of five major parts, including demographic data, dietary data, examination data, laboratory data and questionnaire data. More detailed information on NHANES is available on the official website.

### Participants selection

We performed secondary data analysis based on data of two 2-year NHANES survey cycles: 1999–2000 and 2001–2002. After a series of screenings, we finally selected 6468 out of 21,004 (1999–2000: 9965 cases; 2001–2002: 11039 cases) participants for the final data analysis. We screen participants according to the exclusion criteria listed below: (1) people aged < 18 years (*n* = 10,151); (2) subjects without telomere data (*n* = 3026); (3) use statins [*n* = 704 (atorvastin calcium = 308, cerivastatin sodium = 17, fluvastin sodium = 37, lovastatin = 40, nystatin = 4, pravastatin sodium = 82, simvastatin = 216)]; (4) any cancer or malignancy (*n* = 642); (5) probable phenotypic familial hypercholesterolaemia (FH) (*n* = 13). (Additional file 1: Figure S1). We noted that the research is not a clinical trial and therefore does not need to be registered. The research procedure is in accordance with the Helsinki Declaration of the World Medical Association (See NHANES official website for details.)

### Variables

In the present study, the dependent variables were low density lipoprotein cholesterol (LDL-C, mg/dL), high density lipoprotein cholesterol (HDL-C, mg/dL) and triglyceride (TG, mg/dL), respectively. Targeted independent variable was mean telomere length (T/S ratio).

For covariates, continuous variables consisted of age (year), poverty to income ratio, alanine aminotransferase (ALT, U/L), aspartate aminotransferase (AST, U/L), serum urea nitrogen (BUN, mg/dL), serum creatinine (Cr, mg/dL), serum uric acid (UA, mg/dL), body mass index (BMI, kg/m^2^), dietary interview - individual foods [alcohol (gm), caffeine (mg), calcium (mg), carbohydrate (gm), cholesterol (mg), dietary fiber (gm), energy (kcal), total monounsaturated fatty acids (gm), total polyunsaturated fatty acids (gm), total saturated fatty acids (gm), total fat (gm)] and CRP.

Categorical variables included sex (male, female), race/ethnicity (Mexican American, other hispanic, non-hispanic white, non-hispanic black, other race), educational level (or level of education) (less than high school, high school, more than high school), any diabetes, any hypertension, any coronary atherosclerotic heart disease (CAD), any family CAD, smoking status (current, past, none), marital status (married, single, living with partner), physical activity (sits, walks, light loads, heavy work), In general, the covariates included demographic data, dietary data, physical examination data, and comorbidities, etc. Telomere length, lipoprotein measurement process and other covariate acquisition process are available at http://cdc.gov/nchs/nhanes.

### Definition of familial hypercholesterolaemia

We employed the Dutch Lipid Clinic Network (DLCN) criteria to define FH. The DLCN criteria consisted of five parts, including family history of FH (first-degree relative), personal history of peripheral arterial disease and early ASCVD, physical examination, serum LDL-C testing and pathogenic mutation. However, we only used serum LDL-H level and personal history of early ASCVD to diagnose the FH due to the unavailability of data of family history of hypercholesterolaemia, personal history of peripheral arterial disease, physical stigmata of FH and pathogenic mutation. In DLCN criteria, LDL-C levels graded from 8 points for LDL > 8.5 mmol/L (330 mg/dL) down to 1 point for 4.0–4.9 mmol/L (155–189 mg/dL), personal history of early ASCVD (2 points). We classified subjects as having definite FH (> 8 points), probable FH (6–8 points), possible FH (3–5 points), or unlikely FH (< 3 points).

### Statistical analysis and missing data

Of the 6468 individuals in the analytic sample, we listed the missing data for each variable in Additional file 2: Table S1.

For missing of dependent variable (LDL-C is missing 3426). We did a sensitivity comparative analysis between participants with vs without known LDL-C data (Additional file 2: Table S2). The purpose of this sensitivity analysis was to investigate whether LDL-C missing is random, and whether it would bias our findings [6]. Our results demonstrated that nearly all variables were similar in patients with available data on LDL-C and participants with missing data on LDL-C.

For missing of covariates, we used multiple multivariate imputations. Our purpose was to maximise statistical power and minimise bias that might occur covariates with missing data were excluded from data analyses [7]. We created 5 imputed datasets with chained equations using a Mice software package [8]. In addition, we used sensitivity analysis to identify whether created complete data had significant difference from pre-imputation data (Additional file 2: Table S3). Our findings demonstrated that created complete data showed no significant difference from raw data. Therefore, all results of our multivariable analyses were based on the imputed datasets and were combined with Rubin’s rules.

The statistical analysis was performed according to the guidelines of the CDC (https://wwwn.cdc.gov/nchs/nhanes/tutorials/default.aspx). We accounted for marked variance and used the proposed weighting methodology. Because the distribution of telomere length is mostly below 1 (percentile 5–95% was 0.6715 to 1.5074), therefore, we multiply the telomere length by 10 (per 0.1 change).Continuous variables were expressed as mean ± standard deviation. Categorical variables were expressed in frequency or as a percentage. We used weighted chi-square test (categorical variables) or weighted linear regression model (continuous variables) to calculate the differences among different telomere length groups (quartile). To investigate whether telomere length is correlated with lipoprotein in selected participants, our statistical analysis consisted of three main steps.

Step 1: weighted univariate and multivariate linear regression model were employed. We constructed three models: **model 1**, no covariates were adjusted; **model 2**, only adjusted for sociodemographic data; **model 3,** model 2 + other covariates presented in Table 1.

**Table 1:** Baseline characteristics of participants

Telomere length (R/S ratio)	Q1	Q2	Q3	Q4	*P* value
n	1617	1617	1617	1617	
lipoprotein					
LDL-C, mean ± SD (mg/dL)	128.66 ± 32.64	125.47 ± 33.47	120.82 ± 33.24	120.14 ± 34.41	< 0.0001
HDL-C, mean ± SD (mg/dL)	50.27 ± 14.85	50.67 ± 16.01	50.95 ± 15.26	51.42 ± 15.02	0.1641
Triglycerides, mean ± SD (mg/dL)	159.05 ± 136.78	145.78 ± 106.29	146.08 ± 153.61	129.76 ± 101.80	< 0.0001
Sociodemographic variables					
Age, mean ± SD (years)	54.54 ± 16.91	46.34 ± 15.34	41.57 ± 14.67	37.37 ± 13.04	< 0.0001
Poverty to income ratio, mean ± SD (years)	0.75 ± 0.08	0.93 ± 0.04	1.09 ± 0.05	1.40 ± 0.25	< 0.0001
Sex (%)					0.2236
male	51.04	49.18	47.37	49.19	
female	48.96	50.82	52.63	50.81	
Race/Ethnicity (%)					< 0.0001
Mexican American	7.01	7.84	8.90	6.70	
Non-Hispanic Black	7.94	8.51	9.55	12.92	
Non-Hispanic White	75.75	73.36	69.74	66.70	
Other Hispanic	6.07	6.36	6.69	9.37	
Other race/ethnicity	3.24	3.93	5.13	4.31	
Education (%)					< 0.0001
less than high schoo	27.77	21.22	20.97	17.70	
high school	25.76	26.44	24.83	26.27	
more than high school	46.48	52.34	54.20	56.03	
Martial Status (%)					< 0.0001
married	71.65	65.36	60.13	53.23	
single	23.25	28.38	34.21	39.37	
living with partner	5.10	6.27	5.66	7.40	
Variables of laboratory data					
ALT, mean ± SD (U/L)	26.38 ± 20.63	26.61 ± 21.67	26.68 ± 56.22	26.18 ± 27.74	0.9715
AST, mean ± SD(U/L)	25.68 ± 20.19	24.26 ± 12.58	23.66 ± 11.28	24.65 ± 22.67	0.0096
Blood urea nitrogen, mean ± SD (mg/dL)	15.14 ± 6.26	13.74 ± 4.68	13.34 ± 5.05	13.11 ± 4.44	< 0.0001
Creatinine, mean ± SD(mg/dL)	0.86 ± 0.49	0.83 ± 0.43	0.82 ± 0.39	0.80 ± 0.28	0.0003
C-reactive protein(mg/dL)	0.51 ± 0.88	0.43 ± 0.83	0.40 ± 0.65	0.33 ± 0.56	< 0.0001
Uric acid, mean ± SD(mg/dL)	5.52 ± 1.51	5.32 ± 1.41	5.29 ± 1.48	5.26 ± 1.46	< 0.0001
Medical examination and personal life history					
Body Mass Index, mean ± SD (kg/m^2^)	28.67 ± 6.09	28.25 ± 6.27	27.78 ± 6.14	27.42 ± 6.25	< 0.0001
Physical Activity (MET-based rank) (%)					< 0.0001
Sits	26.29	19.47	19.37	18.22	
Walks	27.42	29.48	25.63	27.99	
Light loads	18.92	18.84	19.68	19.08	
Heavy work	27.36	32.21	35.31	34.72	
Current or Past Cigarette Smoker (%)					< 0.0001
none	47.76	47.87	53.41	53.81	
current	30.24	26.73	21.59	19.15	
past	22.00	25.40	25.00	27.04	
Dietary interview - individual foods					
Alcohol, mean ± SD (gm)	9.31 ± 36.24	10.49 ± 30.58	11.41 ± 32.26	15.07 ± 46.08	< 0.0001
Caffeine, mean ± SD (mg)	220.92 ± 276.11	233.16 ± 325.75	190.05 ± 226.56	179.96 ± 250.56	< 0.0001
Calcium, mean ± SD (mg)	832.32 ± 681.72	847.10 ± 551.24	881.93 ± 613.60	901.43 ± 642.76	0.0053
Carbohydrate, mean ± SD (gm)	258.16 ± 141.13	275.16 ± 136.13	281.59 ± 128.51	295.30 ± 142.53	< 0.0001
Cholesterol, mean ± SD (mg)	274.05 ± 242.38	297.85 ± 242.96	298.35 ± 254.11	293.91 ± 234.86	0.0191
Dietary fiber, mean ± SD (gm)	15.86 ± 11.70	15.71 ± 9.86	15.61 ± 9.61	16.14 ± 10.66	0.4535
Energy, mean ± SD (kcal)	2084.67 ± 1023.12	2241.17 ± 1039.35	2272.85 ± 1018.07	2367.07 ± 1085.04	< 0.0001
Total monounsaturated fatty acids, mean ± SD (gm)	29.81 ± 18.42	32.12 ± 20.16	31.56 ± 19.47	32.15 ± 19.17	0.0021
Total polyunsaturated fatty acids, mean ± SD (gm)	16.14 ± 10.83	17.65 ± 12.43	17.04 ± 11.51	17.39 ± 11.84	0.0024
Total saturated fatty acids, mean ± SD (gm)	25.88 ± 17.18	27.99 ± 16.81	28.04 ± 18.00	28.32 ± 17.37	0.0003
Total fat, mean ± SD (gm)	79.32 ± 46.23	86.01 ± 49.33	84.40 ± 48.42	85.63 ± 48.05	0.0003
Comorbidities (%)					
Any Diabetes (FBG > = 126 mg/dL or self-report)					< 0.0001
No	89.27	93.06	93.87	95.14	
Yes	10.73	6.94	6.13	4.86	
Any Hypertension (BP > = 139/90 or self report)					< 0.0001
No	66.69	76.41	77.14	82.74	
Yes	33.31	23.59	22.86	17.26	
Any CAD (self report)					< 0.0001
No	94.80	97.14	98.13	98.85	
Yes	5.20	2.86	1.87	1.15	
Any family with heart attack or angina					< 0.0001
No	67.61	65.43	60.84	59.47	
Yes	32.39	34.57	39.16	40.53	

Mean +/− SD for continuous variables: *P* value was calculated by weighted linear regression model

% for Categorical variables: *P* value was calculated by weighted chi-square test

Step 2: To address for nonlinearity of lipoproteins and telomere lengths, a weighted generalized additive model (GAM) and smooth curve fitting (penalized spline method) were conducted. If nonlinearity was detected, we firstly calculated the inflection point using recursive algorithm, and then constructed a weighted two-piecewise linear regression model on both sides of the inflection point. We determined the best fit model (linear regression model vs two-piecewise linear regression model) based on the *P* values for log likelihood ratio test.

Step 3: The subgroup analyses were performed using weighted stratified linear regression models. For continuous variable, we firstly converted it to a categorical variable according to the clinical cut point or tertile, and then performed an interaction test. Tests for effect modification by subgroup used interaction terms between subgroup indicators, followed by the likelihood ration test.

To ensure the robustness of data analysis, we did the following sensitivity analysis: (1) we converted the telomere length into a categorical variable by quartile, and calculated the P for trend. The purpose was to verify the results of telomere length as a continuous variable and to observe the possibility of nonlinearity. 2) using the weighted GAM model to adjust the continuous variables in the model 3.

All the analyses were performed with the statistical software packages R (http://www.R-project.org, The R Foundation) and EmpowerStats (http://www.empowerstats.com, X&Y Solutions, Inc., Boston, MA). *P* values less than 0.05 (two-sided) were considered statistically significant.

## Results

### Baseline characteristics of participants

The weighted distribution of selective participants sociodemographic characteristics and other covariates for the selected NHANES 1999 to 2002 population is shown in Table 1. The average age of the participants was 47.4 ± 18.4 (19–85) years old, and about 52.1% of them were female. Among different groups of telomere length (quartile, Q1-Q4), the distributions by HDL-C, sex, ALT and dietary fiber intake are similar. Compared with Q1 and Q2 groups, participants with higher telomere length were younger, had a higher poverty to income ratio, education and single persons in Q3 and Q4 groups. On the contrary, Q3 and Q4 groups had lower LDL-C, TG, BMI and current smokers.

### Telomere length and lipoproteins

We listed effect sizes of association between lipoproteins and telomere length in Table 2. These effect sizes of model 2, 3 and GAM models were pooled by Rubin’s rule using imputation data (see Additional file 2: Tables S4, S5 and S6 for details). Model 1 is an unadjusted model. This model indicated that telomere length were negatively associated with TG and LDL-C, and positively associated with HDL-C. These results were verified by sensitivity analysis. In Model 2, after adjusting for sociodemographic variables (age, poverty to income ratio, sex, race/ethnicity, education level, marital status), the association between TG, LDL-C and telomere length were not significant, while HDL-C still showed the positive correlation with telomere length. The similar results can be detected in model 3 (fully-adjusted model). When we adjusted all covariates presented in Table 1, LDL-C and TG was not associated with telomere length, while a difference in 0.1 of telomere length is associated with 0.19 difference in HDL-C. To address for nonlinearity, we also used GAM to adjust continuous variables in covariates. Despite these transformations (fitting continuous variables as smooth), the results did not change significantly (Model 4).

**Table 2:** Association of mean Telomere Length with lipoproteins

Exposure	Model1 β (95% CI)	Model 2 β^a^ (95% CI)	Model 3 β^a^ (95% CI)	GAM model β^a^ (95% CI)
LDL-cholesterol (mg/dL) (*n* = 3042)				
Telomere length (per 0.1 change)	−1.18 (−1.62, − 0.75)	− 0.01 (− 0.48, 0.45)	0.18 (−0.26, 0.68)	0.07 (− 0.35, 0.59)
Telomere length (per 0.1 change)(quartile)				
Q1	ref	ref	ref	ref
Q2	−3.19 (−6.63, 0.25)	0.32 (−3.07, 3.71)	0.02 (−3.32, 3.36)	−0.96 (−4.26, 2.34)
Q3	−7.85 (−11.29, −4.40)	− 1.36 (−4.85, 2.14)	−0.87 (− 4.31, 2.58)	−1.28 (− 4.66, 2.11)
Q4	−8.52 (− 11.93, −5.12)	−0.16 (− 3.74, 3.41)	0.81 (−2.72, 4.33)	−0.09 (− 3.59, 3.40)
P for trend	< 0.0001	0.930	0.622	0.873
Triglycerides (mg/dL) (*n* = 6461)				
Telomere length (per 0.1 change)	− 3.52 (− 4.59, − 2.46)	− 1.56 (− 2.68,-0.44)	− 0.98 (− 2.06, 0.10)	− 0.81 (− 1.74, 0.29)
Telomere length (per 0.1 change)(quartile)				
Q1	ref	ref	ref	ref
Q2	− 13.27 (− 22.02, − 4.52)	− 5.12 (− 13.87, 3.63)	−2.64 (− 11.05, 5.77)	−4.78 (− 12.98, 3.42)
Q3	−12.97 (− 21.64, − 4.30)	− 0.46 (− 9.36, 8.43)	1.46 (−7.10, 10.02)	− 0.12 (− 8.48, 8.23)
Q4	−29.28 (− 37.80, − 20.76)	−13.06 (− 22.11, − 4.01)	−8.95 (− 17.66, − 0.23)	−8.19 (− 16.69, 0.31
P for trend	< 0.0001	0.013	0.117	0.082
HDL-Cholesterol (mg/dL) (*n* = 6461)				
Telomere length (per 0.1 change)	0.16 (0.03, 0.29)	0.33 (0.20, 0.46)	0.19 (0.03, 0.37)	0.13 (0.01, 0.33)
Telomere length (per 0.1 change)(quartile)				
Q1	ref	ref	ref	ref
Q2	0.41 (− 0.66, 1.47)	0.93 (− 0.07, 1.94)	0.50 (− 0.42, 1.42)	0.24 (− 0.65, 1.13)
Q3	0.68 (− 0.37, 1.74)	1.49 (0.47, 2.51)	0.95 (0.01, 1.89)	0.79 (− 0.11, 1.70)
Q4	1.15 (0.11, 2.19)	2.60 (1.56, 3.64)	1.48 (0.53, 2.44)	1.15 (0.23, 2.08
P for trend	0.0245	< 0.001	0.001	0.023

95% CI: 95% Confidence interval ^a^:Indicated effect sizes (β) were combined by Rubin’s rule

Model 1: no covariates were adjusted

Model 2: only sociodemographic variables were adjusted (age, poverty to income ratio, sex, race/ethnicity, education level, marital status)

Model 3: all covariates presented in Table 1 were adjusted

Model 4: all continuous variables in the covariates were adjusted as smooth

**Fig. 1:**
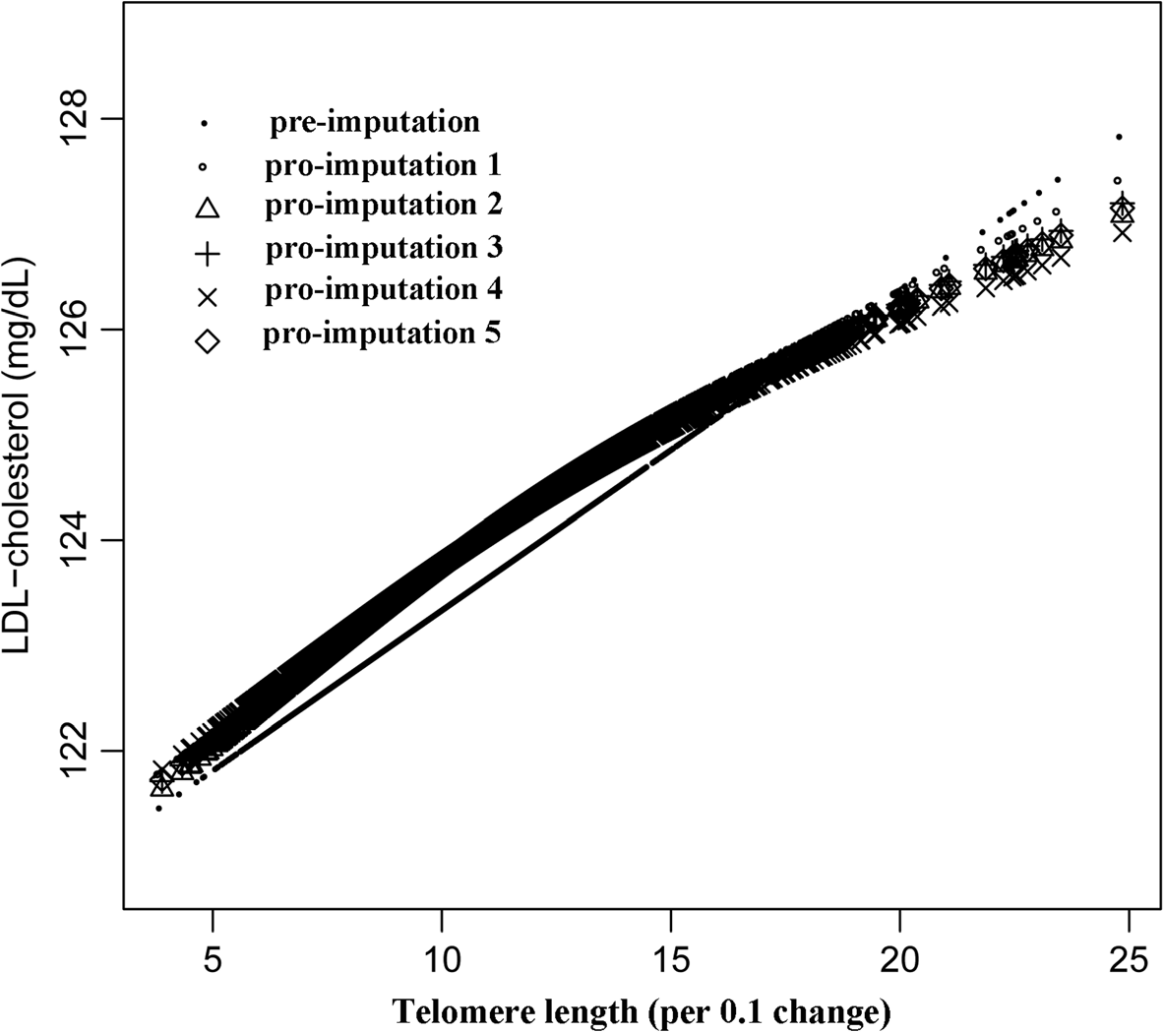
The correlation between LDL-C and telomere length (using penalized spline method). Different line patterns indicated different data sources (pre- or post-imputation)

**Fig. 2:**
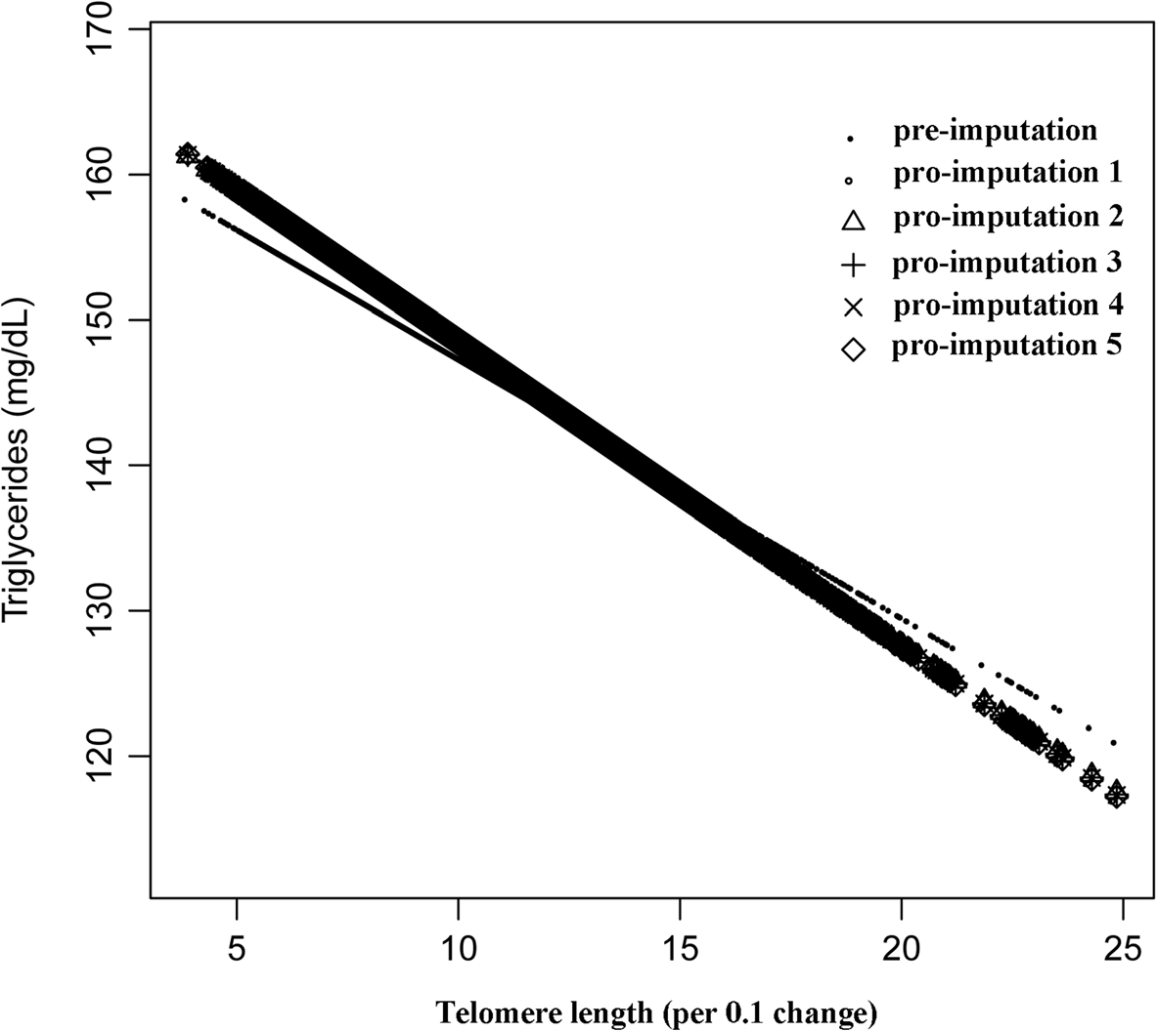
The correlation between triglyceride and telomere length (using penalized spline method). Different line patterns indicated different data sources (pre- or post imputation)

**Fig. 3:**
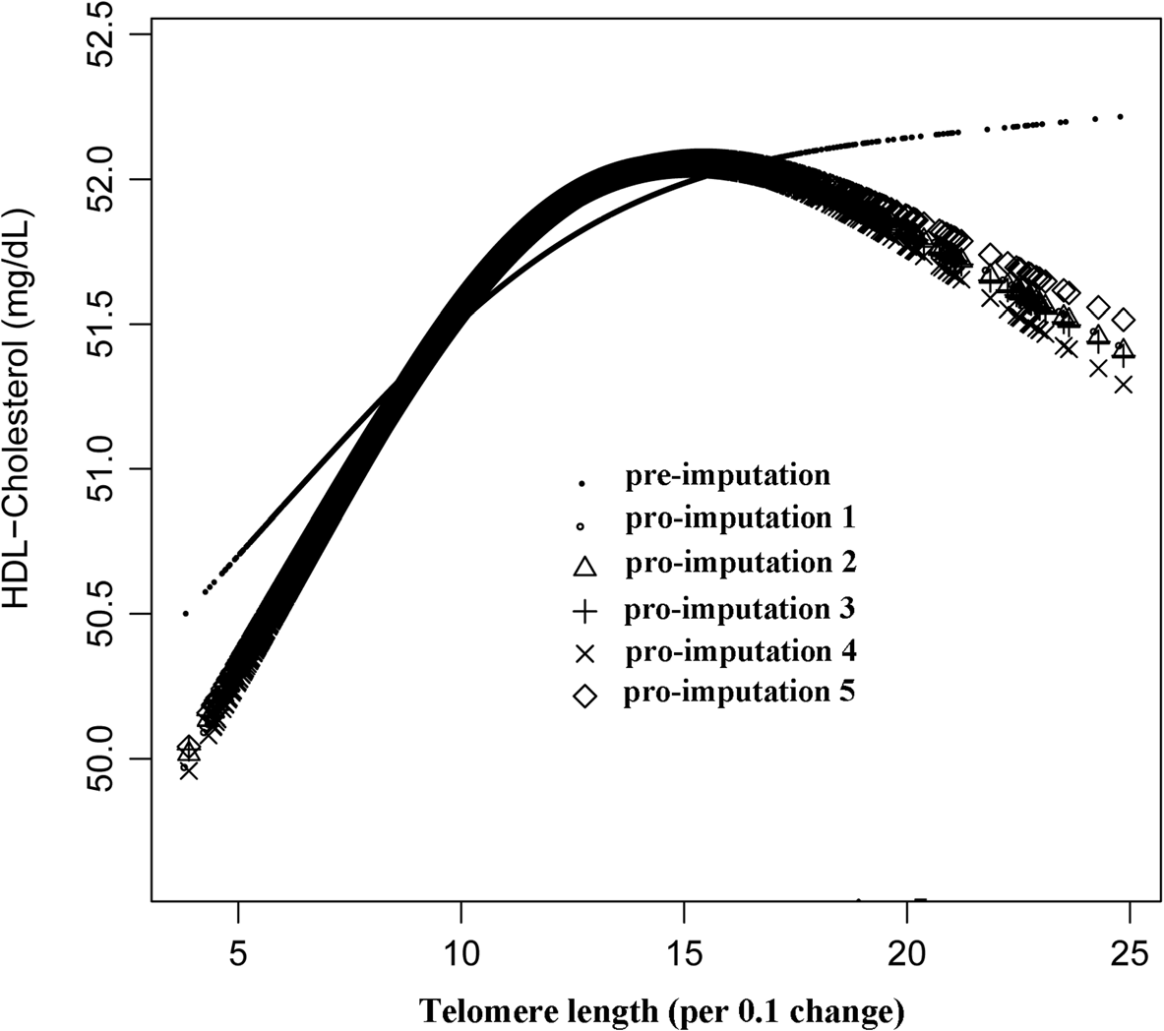
The correlation between HDL-C and telomere length (using penalized spline method). Different line patterns indicated different data sources (pre- or post imputation)

We also tried to use GAM model and penalized spline method to find the nonlinear relationship between lipoproteins and telomere length (Figs. 1, 2 and 3 and Table 3). Because of the limitations of methodology, we were unable to pool the nonlinear relationships among the post-imputation data. In the present study, therefore, we only used the pre-imputation data to explore the nonlinearity of lipoproteis and telomere length. However, we also did a sensitivity analysis, it showed that the nonlinear trends of the data among pre- and post- imputation were approximately the same (Figs. 1, 2 and 3, Additional file 2: Tables S7, S8 and S9). Among lipoproteins, only the relationship between HDL-C and telomere length was nonlinear. We then used a recursive algorithm to calculate the inflection point and fitted the relationship between lipoprotein and telomere length by a weighted two-piecewise linear model. The inflection point we got was 1.25. On the left side of the inflection point (telomere length ≤ 1.25), a difference in 0.1 of telomere length was associated with 0.50 difference in HDL-C. On the right side of the inflection point, we failed to observe a correlation between HDL-C and telomere length (β = − 0.22, 95% CI: -0.53, 0.09).

**Table 3:** Results of standard linear regression model and two-piecewise linear regression model

Outcome:	LDL-cholesterol β (95% CI)	Triglycerides β (95% CI)	HDL-Cholesterol β (95% CI)
Fitting model by standard linear regression	0.21 (− 0.30, 0.72)	−1.42 (−2.75, − 0.09)	0.17 (0.04, 0.31)
Fitting model by two-piecewise linear regression			
Inflection point of telomere length (per 0.1 change)	7.33	7.21	12.52
≤ inflection point	3.11 (−2.21, 8.43)	7.07 (− 9.03, 23.17)	0.50 (0.27, 0.73)
> inflection point	0.22 (−0.32, 0.76)	−1.77 (− 3.21, − 0.32)	−0.22 (− 0.53, 0.09)
P for log likelihood ratio test	0.297	0.291	0.001

All results presented in Table 3 were calculated by pre-imputation data. All models adjusted the same covariates, including age (Smooth); any CAD (self report); any family with heart attack or angina; sex; poverty to income ratio(Smooth); martial status; race; education; ALT (Smooth); AST (Smooth); blood urea nirogen (Smooth); creatinine (Smooth); Uric acid (Smooth); C reaction protein (smooth); BMI (Smooth); physical activity; current or past cigarette smoker?; alcohol (Smooth); caffeine (Smooth); calcium (Smooth); carbohydrate (Smooth); cholesterol (Smooth); dietary fiber (Smooth); energy (Smooth); total monounsaturated fatty acids (Smooth); total polyunsaturated fatty acids (Smooth); total saturated fatty acids (Smooth); total fat (Smooth); any diabetes; any hypertension

### Subgroup analysis

We tested interactions with all covariates presented in Table 1 (Additional file 2: Tables S10, S11 and S12). Only a small number of interactions were observed. For correlation on LDL-C and telomere length, the tests for interactions were significant for race/ ethnicity (P for interaction = 0.048). Besides, we found that the association of LDL with telomere length was only significant in participants of Non-Hispanic Black. In Non-Hispanic Black, a difference in 0.1 of telomere length is associated with 2.43 differences in LDL-C (95% CI: 0.80–4.05). In addition, we found the positive correlation between LDL-C and telomere length in participants with any hypertension (2.53, 1.41–3.65).

For correlation on HDL-C and telomere length, the test for interactions were significant for smoking status (P for interaction = 0.016). We did not observe a relationship between HDL-C and telomere length in non-smokers and those current smokers. However, among ex-smoker, we found that HDL-C was positively correlated with telomere length.

For correlation on TG and telomere length, the same, we also detected the significant interaction on smoking status. Compared with ex-smoker and non-smoker, the strongest correlation between TG and telomere length was observed on current smoker (β = − 6.08, 95% CI: -9.02 to − 3.13).

## Discussion

Our primary objective was to investigate whether lipoproteins are independent association of telomere length. Given telomere length is associated with cardiovascular disease mortality, therefore, this issue is important because lipoproteins also are the well-documented risk factors of cardiovascular disease. In the present study, we found that, among a large and nationally representative, randomly selected sample of American adults, there are independent correlations between HDL-C and telomere length after accounting for age, sociodemographic factors, liver and renal functions, medical examination and personal life history, health and diarary behaviors, and comorbidities associated with lipoproteins. Besides, we also found the nonlinearity of relationship between HDL-C and telomere length. The positive correlation between HDL-C and telomere length was saturated when the telomere length reached 1.25.

Rehkopf, D. H et al [5] have conducted a pioneering investigation using the same NHANES data. Their results were partly the same as ours. In that study, they reported that HDL-C and triglycerides were associated with telomere length. However, they simultaneously discussed the relationships between 17 variables (biomarkers of CVD) and telomere length. Therefore, they were destined to be unable to formulate a unified adjustment strategy that can satisfy these 17 variables based on clinical experience and previous research. For example, when investigating the relationship between C-reactive protein and telomere length, there was no need to adjust dietary data; but when investigating the relationship between lipoprotein and telomere length, dietary factors should be considered. Interestingly, we also found Rehkopf, D. H has addressed the nonlinearity between HDL-C and telomere length, which was the same as our study. However, due to the different focus of the research, Rehkopf, D. H did not discuss this nonlinear relationship in depth.

According to STROBE statement [9], subgroup analysis can make better use of data to reveal underlying truths. In the present study, we found that smoking status modified the relationship between lipoproteins and telomere length. Some researchers have confirmed that cigarette smoking can accelerate the attrition and therefore biological aging through mechanisms involving oxidative stress [10–12]. In addition, smokers have the abnormal TG tolerance and lower HDL-C [13, 14]. Although the present study is not a mechanism-driven research, it may be used to explain why a stronger association for current smokers for triglycerides, and why the positive correlation with telomere length only can be detected in ex-smokers for HDL-C. However, we also found that the association between telomere length and HDL-C was not detectable in diabetic populations (0.20, − 0.43 to 0.83). Conversely, in non-diabetic populations, telomere length was positively correlated with high density lipoprotein (0.22, 0.07 to 0.36). The same trend in hypertension (0.07, − 0.24 to 0.38) / non-hypertensive population (0.24, 0.08 to 0.40), CAD (0.18, − 1.07 to 1.43) / non-CAD (0.21, 0.06 to 0.35), hyperuricemia (− 0.06, − 0.42 to 0.31)/non-hyperuricemia (0.22, 0.06 to 0.38) was also found. In summary, the association between HDL-C and telomere length was not observed in pathological states (hypertension, diabetes, CAD). The appearance of these results were not accidental. We speculated the disease-specificity of relationship between HDL-C and telomere length may be related to dysfunction of HDL-C in pathological states. Previous study [15] has reported that under pathological states (including diabetes, CAD, hypertension), the function of HDL-C will be abnormal. These findings may be used to explain the disease-specificity of relationship between HDL-C and telomere length.

For LDL-C, our findings suggested participants with any hypertension and Non-Hispanic Black showed the strong and positive correlation with telomere length. A series of population-based studies have demonstrated that shorter telomere length was associated with hypertension [16–18]. Besides, patients with hypertension have the higher LDL-C than those non-hypertension individuals [19, 20]. These evidences may be used to explain our findings about the strong associated for LDL-C in hypertension. However, we cannot explain this racially specific change in LDL-C.

There are some strengthens in the present study. Firstly, we did not only consider the impact of missing data on the results, but also took different treatments based on the type of missing data. Secondly, sensitivity analyses were conducted on missing data and evaluation of effect sizes. Thirdly, we used GAM model to address nonlinearity. Fourthly, saturation effect was observed between HDL-C and telomere length by two-piecewise linear models. It made our conclusion was more valuable and significant in clinical and application compared with previous similar findings. Finally, because this study only focused on investigating the relationship between lipoprotein and telomere length, our adjustment strategy was more adequate.

There are some limitations in our study. Firstly, due to the nature of cross-sectional study, we provided only weak evidence between lipoproteins and telomere length, and it was impossible to obtain the causal inference. Experimental study designs were therefore more adequate to address this issue. Secondly, we excluded participants with familial hypercholesterolemia, any cancer or malignancy, and statin users because these special populations have a great influence on lipoprotein levels or telomere length [12, 21–23]. Therefore, the results obtained in this study cannot be used in the above population. Thirdly, we did not concern about the pregnant population. In the NHANES official website data, pregnancy-related data is largely missing (cycle 1999–2000, missing 8003; cycle 2001–2002, missing 7492). Therefore, simply excluding pregnant people will inevitably lead to bias. Fourthly, we did not adjust white blood cells and constituent cell types. In the study of Rehkopf, D. H, et al., argued that the reason for the adjustments of white blood cells and constituent cell types is that telomere length comes from a mix of blood types that have systematically different extents of differentiation depending on function and thus have different mean telomere lengths. However, measurement error is not equal to measurement bias, and it is reasonable to assume that the potential measurement errors will not bias our findings. Furthermore, there are some studies on telomere length using NHANES data do not adjust constituent cell types as well [21, 24–27]. Finally, NHANES survey itself is a cross-sectional study and does not contain other drug information that may interfere with lipid metabolism. Therefore, the bias caused by reverse epidemiology and unmeasurable confounding factors are not excluded. Besides, it would be useful to carefully record the medication history as a potential confounding factor in future studies.

## Conclusion

After adjusting for demographic data, dietary data, physical examination data, and comorbidities, telomere length is not associated with LDL-C and TG. The relationship between HDL-C and telomere length is non-linear. Telomere length is positively associated with HDL-C when telomere length is less than 1.25.

## Additional files

Additional file 1: The flowchat of participants selection. (TIF 20853 kb)

Additional file 2: The missing data description, multiple imputation and effect value pool details and interaction test analysis. (DOC 775 kb)

## Abbreviations

HDL-C: High-density lipoprotein cholesterol
LDL: Low density lipoprotein
TC: Total cholesterol
