# Supplementary figures and images for: Association between lipoproteins and telomere length in US adults: data from the NHANES 1999–2002
Source: Lipids Health Dis. 2019 Apr 1;18:80. doi: 10.1186/s12944-019-1030-7 (PMC6444542; doi:10.1186/s12944-019-1030-7)

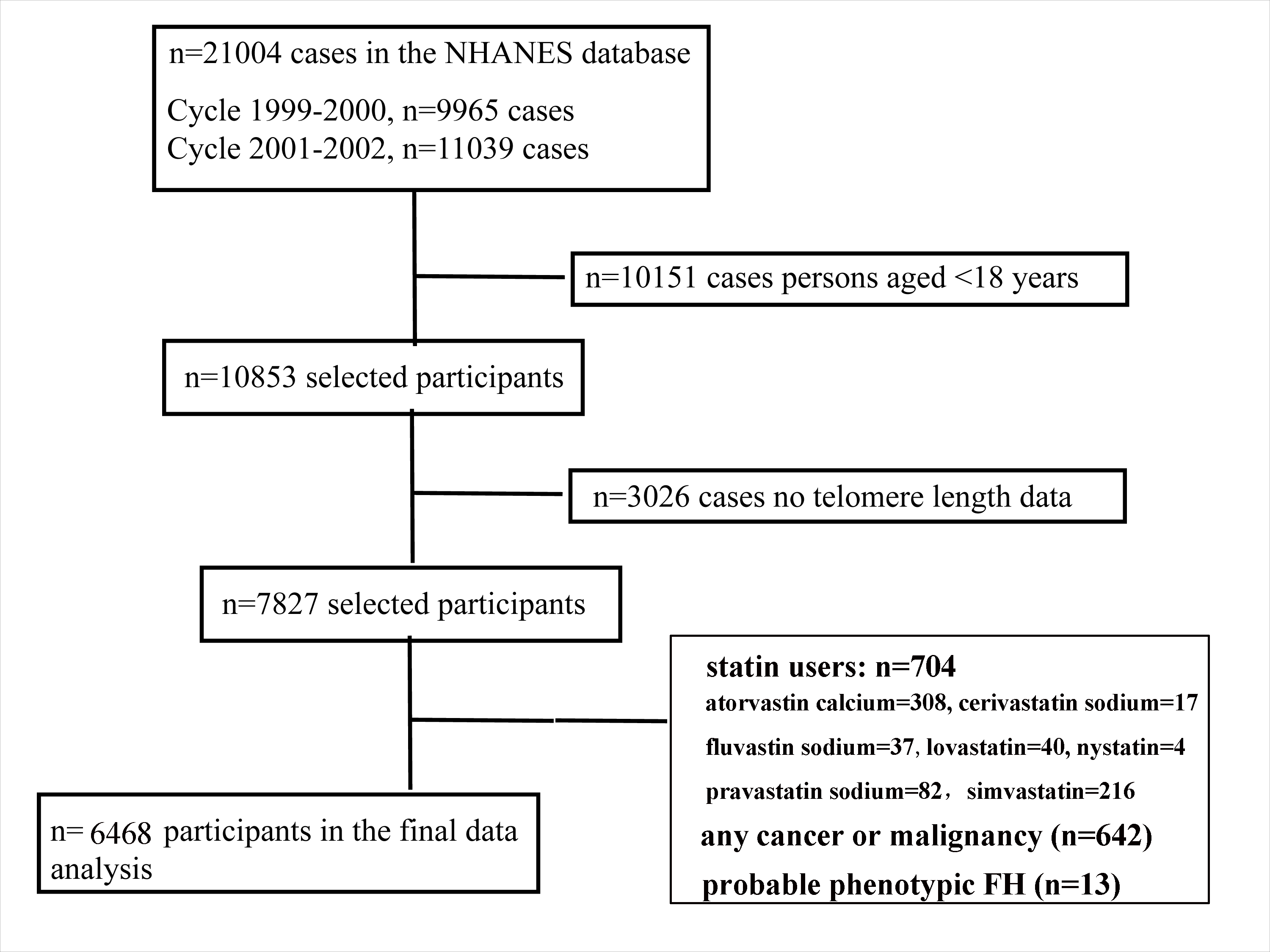

Supplement: Supplementary file 1 — The flowchat of participants selection. (TIF 20853 kb) [file 12944_2019_1030_MOESM1_ESM.tif]
