# Supplementary material for: Association between lipoproteins and telomere length in US adults: data from the NHANES 1999–2002
Source: Lipids Health Dis. 2019 Apr 1;18:80. doi: 10.1186/s12944-019-1030-7 (PMC6444542; doi:10.1186/s12944-019-1030-7)
Supplement: Supplementary file 2 — The missing data description, multiple imputation and effect value pool details and interaction test analysis. (DOC 775 kb) [file 12944_2019_1030_MOESM2_ESM.doc]

**On-line Supporting Material**

Supplement table 1：The description of missing data

| Variables | Non-missing | missing |
| --- | --- | --- |
| ID | 6468 | 0 |
| age (years) | 6468 | 0 |
| sex | 6468 | 0 |
| Education | 6456 | 12 |
| LDL-cholesterol | 3042 | 3426 |
| ALT | 6459 | 9 |
| AST | 6459 | 9 |
| Blood urea nitrogen | 6459 | 9 |
| Triglycerides | 6461 | 7 |
| Uric acid | 6459 | 9 |
| Alcohol intake | 6190 | 278 |
| Caffeine intake | 6190 | 278 |
| Calcium intake | 6190 | 278 |
| Carbohydrate intake | 6190 | 278 |
| Cholesterol intake | 6190 | 278 |
| Dietary fiber intake | 6190 | 278 |
| Energy intake | 6190 | 278 |
| Total monounsaturated fatty acids intake | 6190 | 278 |
| Total polyunsaturated fatty acids intake | 6190 | 278 |
| Total saturated fatty acids intake | 6190 | 278 |
| Total fat intake | 6190 | 278 |
| Creatinine | 6465 | 9 |
| Body Mass Index | 6266 | 202 |
| Physical Activity | 6009 | 459 |
| Any Diabetes | 6406 | 62 |
| Any Hypertension | 6427 | 41 |
| Any CAD | 6429 | 39 |
| Any family with heart attack or angina | 6349 | 119 |
| Current or Past Cigarette Smoker | 6453 | 15 |
| Mean Telomere Length | 6468 | 0 |
| Martial Status | 6116 | 352 |
| Poverty to income ratio | 5848 | 620 |
| HDL-Cholesterol (mg/dL) | 6463 | 5 |
| Race | 6468 | 0 |
| CRP | 6468 | 0 |

Supplement table 2: Sensitivity comparative analysis between participants with vs without known LDL-C data

|  | Participants with known LDL-C data  N=3042 | Participants with missing LDL-C data  N=3426 | P value |
| --- | --- | --- | --- |
| Age (years) | 44.15 ± 16.00 | 44.45 ± 16.28 | 0.4352 |
| ALT (U/L) | 26.85 ± 46.81 | 26.15 ± 22.29 | 0.4064 |
| AST (U/L) | 24.70 ± 20.26 | 24.37 ± 14.79 | 0.4455 |
| Blood urea nitrogen (mg/dL) | 13.57 ± 5.03 | 13.90 ± 5.23 | 0.0085 |
| Triglycerides (mg/dL) | 124.11 ± 66.23 | 159.90 ± 156.05 | <0.0001 |
| Uric acid (mg/dL) | 5.38 ± 1.44 | 5.30 ± 1.48 | 0.0200 |
| Alcohol intake (gm) | 11.47 ± 31.18 | 11.98 ± 41.33 | 0.5816 |
| Caffeine intake(mg) | 212.97 ± 298.59 | 197.69 ± 247.76 | 0.0225 |
| Calcium intake(mg) | 861.73 ± 620.48 | 873.34 ± 624.76 | 0.4497 |
| Carbohydrate intake (gm) | 279.43 ± 132.94 | 278.32 ± 141.45 | 0.7446 |
| Cholesterol intake (mg) | 294.29 ± 243.21 | 289.81 ± 244.10 | 0.4555 |
| Dietary fiber intake (gm) | 15.90 ± 10.68 | 15.78 ± 10.24 | 0.6649 |
| Energy intake (kcal) | 2252.82 ± 1002.21 | 2249.75 ± 1083.59 | 0.9053 |
| Total monounsaturated fatty acids intake (gm) | 31.61 ± 18.75 | 31.40 ± 19.84 | 0.6580 |
| Total polyunsaturated fatty acids intake (gm) | 17.34 ± 11.47 | 16.90 ± 11.90 | 0.1228 |
| Total saturated fatty acids intake (gm) | 27.47 ± 16.87 | 27.79 ± 17.78 | 0.4552 |
| Total fat intake(gm) | 84.32 ± 46.51 | 83.84 ± 49.43 | 0.6854 |
| Creatinine (mg/dL) | 0.82 ± 0.37 | 0.83 ± 0.42 | 0.2425 |
| Body Mass Index (kg/m**2) | 27.80 ± 6.06 | 28.13 ± 6.32 | 0.0344 |
| Mean Telomere Length | 1.06 ± 0.27 | 1.08 ± 0.28 | 0.0084 |
| Poverty to income ratio | 3.02 ± 1.62 | 2.95 ± 1.65 | 0.1057 |
| Direct HDL-Cholesterol (mg/dL) | 50.72 ± 14.84 | 50.98 ± 15.65 | 0.4767 |
| Sex (%) |  |  | 0.4143 |
| male | 49.66 | 48.67 |  |
| female | 50.34 | 51.33 |  |
| Education (< HS, =HS, >HS) (%) |  |  | 0.6823 |
| less than high schoo | 21.22 | 21.81 |  |
| high school | 26.30 | 25.46 |  |
| more than high school | 52.48 | 52.73 |  |
| Physical Activity (MET-based rank) (%) |  |  | 0.0003 |
| Sits | 18.28 | 22.29 |  |
| Walks | 28.79 | 26.70 |  |
| Light loads | 18.70 | 19.49 |  |
| Heavy work | 34.22 | 31.51 |  |
| Any Diabetes (FBG >= 126 mg/dL or self-report) (%) |  |  | 0.1882 |
| No | 93.51 | 92.70 |  |
| Yes | 6.49 | 7.30 |  |
| Any Hypertension? (BP >= 139/90 or self report) (%) |  |  | 0.4041 |
| No | 76.81 | 75.95 |  |
| Yes | 23.19 | 24.05 |  |
| Any CAD (self report) (%) |  |  | 0.2943 |
| No | 97.61 | 97.21 |  |
| Yes | 2.39 | 2.79 |  |
| Any family with heart attack or angina (%) |  |  | 0.0975 |
| No | 61.93 | 63.88 |  |
| Yes | 38.07 | 36.12 |  |
| Current or Past Cigarette Smoker? (%) |  |  | 0.0095 |
| None | 49.27 | 52.26 |  |
| current | 25.69 | 22.71 |  |
| past | 25.04 | 25.03 |  |
| Martial Status (%) |  |  | 0.0011 |
| married | 60.46 | 57.19 |  |
| single | 33.13 | 37.37 |  |
| Living with partner | 6.41 | 5.44 |  |
| Race/Ethnicity (%) |  |  | 0.427 |
| Mexican American | 7.85 | 7.44 |  |
| Non-Hispanic Black | 9.94 | 9.86 |  |
| Non-Hispanic White | 71.53 | 70.69 |  |
| Other Hispanic | 6.58 | 7.74 |  |
| Other race/ethnicity | 4.11 | 4.27 |  |

Nearly all variables were similar in patients with available data on LDL-C and the 3895 participants with missing data on LDL-C.

Supplement table 3：Sensitivity comparative analysis between pre-imputation and post-imputation

|  | Pre-imputation | imputation 1 | imputation 2 | imputation 3 | imputation 4 | imputation 5 | P-value |
| --- | --- | --- | --- | --- | --- | --- | --- |
| ALT | 25.97 ± 33.12 | 25.98 ± 33.11 | 25.98 ± 33.13 | 25.98 ± 33.13 | 25.98 ± 33.11 | 25.99 ± 33.11 | 1.000 |
| AST | 24.67 ± 18.23 | 24.68 ± 18.23 | 24.67 ± 18.24 | 24.67 ± 18.23 | 24.68 ± 18.23 | 24.68 ± 18.22 | 1.000 |
| Blood urea nitrogen | 13.93 ± 5.98 | 13.93 ± 5.98 | 13.93 ± 5.98 | 13.93 ± 5.98 | 13.93 ± 5.97 | 13.93 ± 5.97 | 1.000 |
| Uric acid | 5.31 ± 1.51 | 5.31 ± 1.51 | 5.31 ± 1.51 | 5.31 ± 1.51 | 5.31 ± 1.51 | 5.31 ± 1.51 | 1.000 |
| Alcohol intake | 9.89 ± 33.54 | 10.02 ± 33.64 | 9.93 ± 33.72 | 9.88 ± 33.57 | 9.88 ± 33.48 | 10.07 ± 33.56 | 0.999 |
| Caffeine intake | 164.81 ± 233.00 | 163.26 ± 232.81 | 163.99 ± 232.72 | 163.83 ± 232.91 | 163.82 ± 232.58 | 164.80 ± 232.48 | 0.999 |
| Calcium intake | 817.00 ± 589.77 | 816.70 ± 587.89 | 816.34 ± 587.29 | 818.17 ± 587.99 | 816.10 ± 586.26 | 815.50 ± 587.45 | 1.000 |
| Carbohydrate intake | 268.04 ± 135.72 | 267.87 ± 136.52 | 268.13 ± 135.55 | 267.97 ± 135.30 | 268.40 ± 135.15 | 268.65 ± 134.91 | 0.999 |
| Cholesterol intake | 292.54 ± 245.56 | 293.38 ± 244.91 | 292.68 ± 244.77 | 292.85 ± 245.28 | 292.91 ± 244.60 | 291.80 ± 244.50 | 1.000 |
| Dietary fiber intake | 16.06 ± 10.65 | 16.04 ± 10.68 | 16.08 ± 10.66 | 16.07 ± 10.66 | 16.09 ± 10.65 | 16.08 ± 10.62 | 1.000 |
| Energy intake | 2137.76 ± 1036.01 | 2138.71 ± 1030.76 | 2139.12 ± 1030.04 | 2137.96 ± 1027.42 | 2140.47 ± 1026.50 | 2140.98 ± 1024.22 | 1.000 |
| Total monounsaturated fatty acids intake | 29.52 ± 18.83 | 29.53 ± 18.67 | 29.55 ± 18.69 | 29.53 ± 18.69 | 29.54 ± 18.64 | 29.50 ± 18.59 | 1.000 |
| Total polyunsaturated fatty acids intake | 16.24 ± 11.36 | 16.24 ± 11.32 | 16.26 ± 11.30 | 16.20 ± 11.31 | 16.26 ± 11.31 | 16.25 ± 11.28 | 1.000 |
| Total saturated fatty acids intake | 25.68 ± 16.81 | 25.69 ± 16.64 | 25.68 ± 16.67 | 25.69 ± 16.69 | 25.70 ± 16.64 | 25.65 ± 16.62 | 1.000 |
| Total fat intake | 78.77 ± 46.93 | 78.78 ± 46.48 | 78.83 ± 46.53 | 78.77 ± 46.59 | 78.85 ± 46.44 | 78.72 ± 46.33 | 1.000 |
| Creatinine | 0.82 ± 0.49 | 0.82 ± 0.49 | 0.82 ± 0.49 | 0.82 ± 0.49 | 0.82 ± 0.49 | 0.82 ± 0.49 | 1.000 |
| Body Mass Index | 28.31 ± 6.16 | 28.29 ± 6.15 | 28.30 ± 6.18 | 28.28 ± 6.16 | 28.31 ± 6.18 | 28.30 ± 6.16 | 1.000 |
| Poverty to income ratio | 2.62 ± 1.61 | 2.60 ± 1.62 | 2.60 ± 1.62 | 2.60 ± 1.62 | 2.60 ± 1.62 | 2.59 ± 1.62 | 0.951 |
| Education |  |  |  |  |  |  | 1.000 |
| less than high school | 2230 (34.54%) | 2236 (34.57%) | 2234 (34.54%) | 2233 (34.53%) | 2235 (34.55%) | 2236 (34.57%) |  |
| high school | 1474 (22.83%) | 1476 (22.82%) | 1481(22.90%) | 1477 (22.83%) | 1478 (22.85%) | 1475 (22.80%) |  |
| more than high school | 2752 (42.63%) | 2756 (42.61%) | 2753 (42.56%) | 2758 (42.64%) | 2755 (42.60%) | 2757 (42.63%) |  |
| Physical Activity |  |  |  |  |  |  | 0.866 |
| Sits | 1678 (27.92%) | 1762 (27.24%) | 1795 (27.75%) | 1788 (27.64%) | 1787 (27.63%) | 1806 (27.92%) |  |
| Walks | 1639 (27.28%) | 1772 (27.40%) | 1747 (27.01%) | 1770 (27.37%) | 1781 (27.54%) | 1754 (27.12%) |  |
| Light loads | 1022 (17.01%) | 1094 (16.91%) | 1115 (17.25%) | 1098 (16.98%) | 1100 (17.00%) | 1098 (16.98%) |  |
| Heavy work | 1670 (27.79%) | 1840 (28.45%) | 1811 (27.99%) | 1812 (28.01%) | 1800 (27.83%) | 1810 (27.98%) |  |
| Any Diabetes |  |  |  |  |  |  | 1.000 |
| No | 5758 (89.88%) | 5811 (89.84%) | 5809 (89.81%) | 5805 (89.75%) | 5809 (89.81%) | 5802 (89.79%) |  |
| Yes | 648 (10.12%) | 657 (10.16%) | 659 (10.19%) | 663 (10.25%) | 659 (10.19%) | 666 (10.21%) |  |
| Any CAD |  |  |  |  |  |  | 1.000 |
| No | 6226 (96.84%) | 6260 (96.79%) | 6712 (96.76%) | 6715 (96.80%) | 6715 (96.80%) | 6712 (96.76%) |  |
| Yes | 203 ( 3.16%) | 208 ( 3.21%) | 225 ( 3.24%) | 222 ( 3.20%) | 222 ( 3.20%) | 225 ( 3.24%) |  |
| Any family with heart attack or angina |  |  |  |  |  |  | 1.000 |
| No | 4517 (66.25%) | 4599 (66.30%) | 4592 (66.20%) | 4593 (66.21%) | 4603 (66.35%) | 4601 (66.33%) |  |
| Yes | 2301 (33.75%) | 2338 (33.70%) | 2345 (33.80%) | 2344 (33.79%) | 2334 (33.65%) | 2336 (33.67%) |  |
| Martial Status |  |  |  |  |  |  | 0.663 |
| married | 3789 (57.54%) | 4073 (58.71%) | 3982 (57.41%) | 4040 (58.24%) | 4011 (57.82%) | 3789 (54.62%) |  |
| single | 2412 (36.63%) | 2452(35.35%) | 2515 (36.25%) | 2501 (36.05%) | 2527(36.43%) | 2743 (39.54%) |  |
| living with partner | 384 ( 5.83%) | 412 ( 5.94%) | 440 ( 6.34%) | 396 ( 5.71%) | 399 ( 5.75%) | 405 ( 5.84%) |  |
| Current or Past Cigarette Smoker |  |  |  |  |  |  | 1.000 |
| None | 3639 (52.57%) | 3651 (52.63%) | 3644 (52.53%) | 3649 (52.60%) | 3643 (52.52%) | 3643 (52.52%) |  |
| current | 1727 (24.95%) | 1729 (24.92%) | 1732 (24.97%) | 1732 (24.97%) | 1733 (24.98%) | 1734 (25.00%) |  |
| past | 1556 (22.48%) | 1557 (22.44%) | 1561 (22.50%) | 1556 (22.43%) | 1561 (22.50%) | 1560 (22.49% |  |

Supplement table 4: Results of multivariate linear regression among pre- and post-imputation data (LDL-C vs telomere length)

| Model 2 | Pre-imputation  β,(95%CI) | Pro-imputation1  β,(95%CI) | Pro-imputation2  β,(95%CI) | Pro-imputation3  β,(95%CI) | Pro-imputation4  β,(95%CI) | Pro-imputation5  β,(95%CI) | Pooled by rubin’s rule  β,(95%CI) |
| --- | --- | --- | --- | --- | --- | --- | --- |
| Telomere length  (per 0.1 chage) | 0.05 (-0.44, 0.55) | -0.00 (-0.47, 0.46) | -0.00 (-0.47, 0.46) | -0.02 (-0.49, 0.44) | -0.03 (-0.49, 0.44) | -0.02 (-0.49, 0.44) | -0.01 (-0.48, 0.45) |
| Q1 | ref | ref | ref | ref | ref | ref | ref |
| Q2 | 0.58 (-2.95, 4.11) | 0.34 (-3.05, 3.73) | 0.38 (-3.01, 3.76) | 0.28 (-3.11, 3.67) | 0.33 (-3.06, 3.72) | 0.26 (-3.13, 3.65) | 0.32 (-3.07, 3.71) |
| Q3 | -1.66 (-5.36, 2.03) | -1.37 (-4.86, 2.13) | -1.24 (-4.73, 2.25) | -1.44 (-4.93, 2.06) | -1.35 (-4.84, 2.14) | -1.38 (-4.87, 2.12) | -1.36 (-4.85, 2.14) |
| Q4 | 0.33 (-3.46, 4.12) | -0.09 (-3.67, 3.48) | -0.06 (-3.64, 3.51) | -0.22 (-3.79, 3.35) | -0.23 (-3.80, 3.34) | -0.21 (-3.78, 3.37) | -0.16 (-3.74, 3.41) |
| P for trend | 0.8525 | 0.7467 | 0.7661 | 0.6963 | 0.6948 | 0.7116 | 0.9304 |
| Model 3 |  |  |  |  |  |  |  |
| Telomere length  (per 0.1 chage) | 0.31 (-0.21, 0.82) | 0.18 (-0.28, 0.64) | 0.19 (-0.27, 0.65) | 0.17 (-0.29, 0.63) | 0.17 (-0.29, 0.63) | 0.18 (-0.28, 0.64) | 0.18 (-0.26, 0.68) |
| Q1 | ref | ref | ref | ref | ref | ref | ref |
| Q2 | -0.40 (-4.09, 3.30) | 0.04 (-3.30, 3.39) | 0.14 (-3.20, 3.48) | 0.15 (-3.20, 3.50) | 0.16 (-3.19, 3.50) | 0.09 (-3.26, 3.43) | 0.14 (-3.21, 3.48) |
| Q3 | -0.73 (-4.59, 3.13) | -0.72 (-4.16, 2.72) | -0.64 (-4.08, 2.81) | -0.76 (-4.21, 2.69) | -0.67 (-4.12, 2.77) | -0.70 (-4.14, 2.75) | -0.69 (-4.14, 2.76) |
| Q4 | 2.01 (-1.97, 5.99) | 1.02 (-2.50, 4.55) | 1.06 (-2.47, 4.58) | 0.95 (-2.58, 4.48) | 0.99 (-2.54, 4.51) | 0.97 (-2.56, 4.50) | 0.99 (-2.54, 4.52) |
| P for trend | 0.3429 | 0.6532 | 0.6451 | 0.7037 | 0.6807 | 0.6780 | 0.674 |
| GAM model |  |  |  |  |  |  |  |
| Telomere length  (per 0.1 chage) | 0.18 (-0.33, 0.69) | 0.01 (-0.45, 0.46) | 0.05 (-0.41, 0.50) | 0.06 (-0.39, 0.52) | 0.05 (-0.41, 0.50) | 0.06 (-0.40, 0.51) | 0.07 (-0.35, 0.59) |
| Q1 | ref | ref | ref | ref | ref | ref | ref |
| Q2 | -1.66 (-5.30, 1.99) | -1.20 (-4.50, 2.10) | -1.11 (-4.41, 2.20) | -0.93 (-4.23, 2.37) | -0.86 (-4.17, 2.45) | -0.90 (-4.21, 2.42) | -0.95 (-4.26, 2.36) |
| Q3 | -1.41 (-5.20, 2.37) | -1.24 (-4.62, 2.13) | -1.38 (-4.76, 2.01) | -1.11 (-4.50, 2.28) | -0.98 (-4.37, 2.41) | -1.18 (-4.58, 2.21) | -1.16 (-4.57, 2.24) |
| Q4 | 0.84 (-3.09, 4.77) | -0.36 (-3.84, 3.12) | 0.00 (-3.48, 3.48) 0 | -0.00 (-3.49, 3.48) | 0.17 (-3.32, 3.65) | 0.04 (-3.46, 3.53) | 0.05 (3.44, 3.54) |
| P for trend | 0.5918 | 0.887*8* | 0.9288 | 0.9811 | 0.9019 | 0.9791 | 0.950 |

95%CI: 95% Confidence interval

Model 2: only sociodemographic variables were adjusted ( age, poverty to income ratio, sex, race/ethnicity, education level, marital status)

Model 3: all covariates presented in table 1 were adjusted

Model 4: all continuous variables in the covariates were adjusted as smooth

Supplement table 5: Results of multivariate linear regression among pre- and post-imputation data (Triglycerides vs telomere length)

| Model 2 | Pre-imputation  β,(95%CI) | Pro-imputation1  β,(95%CI) | Pro-imputation2  β,(95%CI) | Pro-imputation3  β,(95%CI) | Pro-imputation4  β,(95%CI) | Pro-imputation5  β,(95%CI) | Pooled by rubin’s rule  β,(95%CI) |
| --- | --- | --- | --- | --- | --- | --- | --- |
| Telomere length  (per 0.1 chage) | -1.15 (-2.36, 0.07) | -1.57 (-2.69, -0.45) | -1.56 (-2.69, -0.44) | -1.56 (-2.68, -0.44) | -1.55 (-2.68, -0.43) | -1.55 (-2.67, -0.43) | -1.56 (-2.68,-0.44) |
| Q1 | ref | ref | ref | ref | ref | ref | ref |
| Q2 | -4.37 (-13.76, 5.03) | -5.17 (-13.92, 3.58) | -5.14 (-13.89, 3.61) | -5.04 (-13.79, 3.71) | -5.12 (-13.87, 3.62) | -5.12 (-13.87, 3.63) | -5.12 (-13.87, 3.63) |
| Q3 | 1.08 (-8.55, 10.71) 0 | -0.54 (-9.43, 8.36) | -0.56 (-9.45, 8.34) 0. | -0.43 (-9.33, 8.47) | -0.37 (-9.27, 8.53) | -0.42 (-9.32, 8.47) | -0.46 (-9.36, 8.43) |
| Q4 | -10.36 (-20.19, -0.53) | -13.15 (-22.20, -4.10) | -13.09 (-22.14, -4.04) | -12.99 (-22.04, -3.93) | -13.02 (-22.07, -3.97) | -13.06 (-22.11, -4.00) | -13.06 (-22.11, -4.01) |
| P for trend | 0.0926 | 0.0130 | 0.0133 | 0.0141 | 0.0142 | 0.0138 | 0.0133 |
| Model 3 |  |  |  |  |  |  |  |
| Telomere length  (per 0.1 chage) | -1.15 (-2.52, 0.21) | -1.02 (-2.10, 0.06) | -0.99 (-2.07, 0.10) | -1.00 (-2.09, 0.08) | -0.98 (-2.06, 0.10) | -0.99 (-2.07, 0.09) | -1.15 (-2.52, 0.21) |
| Q1 | ref | ref | ref | ref | ref | ref | ref |
| Q2 | -3.82 (-13.83, 6.19) | -2.48 (-10.89, 5.93) | -3.03 (-11.44, 5.37) | -2.69 (-11.09, 5.72) | -2.68 (-11.09, 5.73) | -2.64 (-11.04, 5.77) | -2.76 (-11.17, 5.65) |
| Q3 | 2.29 (-7.97, 12.55) | 1.63 (-6.94, 10.19) | 1.41 (-7.15, 9.97) | 1.49 (-7.07, 10.06) | 1.59 (-6.98, 10.16) | 1.79 (-6.77, 10.35) | 1.57 (-6.99, 10.14) |
| Q4 | -9.07 (-19.59, 1.44) | -9.17 (-17.88, -0.46) | -9.24 (-17.95, -0.53) | -9.32 (-18.03, -0.61) | -9.04 (-17.75, -0.32) | -9.03 (-17.74, -0.32) | -9.16 (-17.87, -0.44) |
| P for trend | 0.1931 | -0.0728 | 0.0765 | 0.0691 | 0.0808 | 0.0820 | 0.077 |
| GAM model |  |  |  |  |  |  |  |
| Telomere length  (per 0.1 chage) | -1.29 (-2.62, 0.04) | -0.88 (-1.94, 0.17) | -0.81 (-1.86, 0.25) | -0.83 (-1.89, 0.23) | -0.75 (-1.81, 0.30) | -0.80 (-1.86, 0.25) | -0.81 (-1.74, 0.29) |
| Q1 | ref | ref | ref | ref | ref | ref | ref |
| Q2 | -7.93 (-17.74, 1.89) | -5.08 (-13.29, 3.13) | -5.08 (-13.28, 3.13) | -4.92 (-13.13, 3.29) | -5.39 (-13.60, 2.83) | -5.14 (-13.36, 3.07) | -5.13 (-13.35, 3.09) |
| Q3 | -1.92 (-11.95, 8.10) | -0.84 (-9.20, 7.52) | -0.55 (-8.91, 7.80) | -0.35 (-8.70, 8.01) | -0.58 (-8.94, 7.78) | -0.71 (-9.07, 7.64) | -0.55 (-8.90, 7.81) |
| Q4 | -11.12 (-21.40, -0.84) | -9.78 (-18.28, -1.27) | -9.50 (-18.00, -0.99) | -9.56 (-18.06, -1.05) | -9.12 (-17.63, -0.62) | -9.30 (-17.80, -0.80) | -9.37 (-17.88, -0.86) |
| P for trend | 0.1101 | 0.0609 | 0.0728 | 0.0682 | 0.0949 | 0.0784 | 0.0788 |

95%CI: 95% Confidence interval

Model 2: only sociodemographic variables were adjusted ( age, poverty to income ratio, sex, race/ethnicity, education level, marital status)

Model 3: all covariates presented in table 1 were adjusted

Model 4: all continuous variables in the covariates were adjusted as smooth

Supplement table 6: Results of multivariate linear regression among pre- and post-imputation data (HDL-C vs telomere length)

| Model 2 | Pre-imputation  β,(95%CI) | Pro-imputation1  β,(95%CI) | Pro-imputation2  β,(95%CI) | Pro-imputation3  β,(95%CI) | Pro-imputation4  β,(95%CI) | Pro-imputation5  β,(95%CI) | Pooled by rubin’s rule  β,(95%CI) |
| --- | --- | --- | --- | --- | --- | --- | --- |
| Telomere length  (per 0.1 chage) | 0.33 (0.20, 0.47) | 0.33 (0.20, 0.46) | 0.33 (0.20, 0.45) | 0.33 (0.20, 0.46) | 0.33 (0.20, 0.45) | 0.33 (0.20, 0.46) | 0.33 (0.20, 0.46) |
| Q1 | ref | ref | ref | ref | ref | ref | ref |
| Q2 | 0.88 (-0.19, 1.94) | 0.94 (-0.07, 1.94) | 0.93 (-0.07, 1.93) | 0.91 (-0.09, 1.92) | 0.93 (-0.07, 1.94) | 0.95 (-0.05, 1.95) | 0.93 (-0.07, 1.94) |
| Q3 | 1.46 (0.37, 2.56) | 1.52 (0.50, 2.54) | 1.51 (0.49, 2.53) | 1.48 (0.46, 2.50) | 1.45 (0.43, 2.47) | 1.50 (0.48, 2.52) | 1.49 (0.47, 2.51) |
| Q4 | 2.78 (1.66, 3.89) | 2.60 (1.56, 3.64) | 2.60 (1.56, 3.64) | 2.58 (1.54, 3.62) | 2.59 (1.55, 3.63) | 2.62 (1.58, 3.66) | 2.60 (1.56, 3.64) |
| P for trend | <0.0001 | <0.0001 | <0.0001 | <0.0001 | <0.0001 | <0.0001 | <0.001 |
| Model 3 |  |  |  |  |  |  |  |
| Telomere length  (per 0.1 chage) | 0.19 (0.05, 0.33) | 0.19 (0.07, 0.31) | 0.19 (0.07, 0.30) | 0.18 (0.06, 0.30) | 0.19 (0.07, 0.31) | 0.19 (0.05, 0.33) | 0.19 (0.03, 0.37) |
| Q1 | ref | ref | ref | ref | ref | ref | ref |
| Q2 | 0.37 (-0.68, 1.42) 0 | 0.51 (-0.41, 1.43) | 0.55 (-0.37, 1.47) | 0.50 (-0.42, 1.42) | 0.52 (-0.40, 1.44) | 0.52 (-0.40, 1.44) | 0.52 (-0.40, 1.44 ) |
| Q3 | 0.74 (-0.34, 1.82) | 0.91 (-0.03, 1.84) | 0.95 (0.02, 1.89) | 0.89 (-0.05, 1.83) | 0.88 (-0.06, 1.82) | 0.89 (-0.05, 1.83) | 0.90 (-0.04, 1.84) |
| Q4 | 1.48 (0.37, 2.59) | 1.53 (0.58, 2.49) | 1.58 (0.62, 2.53) | 1.50 (0.54, 2.45) | 1.49 (0.53, 2.44) | 1.53 (0.58, 2.49) | 1.53 (0.57, 2.48) |
| P for trend | 0.0061 | 0.0011 | 0.0008 | 0.0015 | 0.0016 | 0.0011 | 0.0012 |
| GAM model |  |  |  |  |  |  |  |
| Telomere length  (per 0.1 chage) | 0.17 (0.03, 0.31) | 0.15 (0.03, 0.26) | 0.14 (0.02, 0.25) | 0.13 (0.01, 0.24) | 0.13 (0.01, 0.24) | 0.14 (0.03, 0.26) | 0.13 (0.01, 0.33) |
| Q1 | ref | ref | ref | ref | ref | ref | ref |
| Q2 | 0.21 (-0.81, 1.23) | 0.24 (-0.65, 1.14) | 0.31 (-0.58, 1.20) | 0.24 (-0.65, 1.14) | 0.32 (-0.58, 1.21) | 0.29 (-0.61, 1.18) | 0.29 (-0.61, 1.19) |
| Q3 | 0.78 (-0.26, 1.82) | 0.76 (-0.15, 1.67) | 0.81 (-0.10, 1.72) | 0.69 (-0.22, 1.60) | 0.72 (-0.19, 1.63) | 0.75 (-0.16, 1.66) | 0.74 (-0.17, 1.66) |
| Q4 | 1.24 (0.17, 2.31) | 1.10 (0.17, 2.02) | 1.17 (0.25, 2.10) | 1.08 (0.15, 2.00) | 1.04 (0.11, 1.96) | 1.09 (0.17, 2.02) | 1.10 (0.16, 2.03) |
| P for trend | 0.0120 | 0.0093 | 0.0056 | 0.0125 | 0.0181 | 0.0117 | 0.0116 |

95%CI: 95% Confidence interval

Model 2: only sociodemographic variables were adjusted ( age, poverty to income ratio, sex, race/ethnicity, education level, marital status)

Model 3: all covariates presented in table 1 were adjusted

Model 4: all continuous variables in the covariates were adjusted as smooth

Supplement table 7: nonlinearity among pre- and pro-imputation data (LDL-C vs telomere length)

|  | Pre-imputation | Pro-imputation1 | Pro-imputation2 | Pro-imputation3 | Pro-imputation4 | Pro-imputation5 |
| --- | --- | --- | --- | --- | --- | --- |
| Fitting model by standard linear regression | 0.21 (-0.30, 0.72) | 0.02 (-0.43, 0.48) | 0.08 (-0.38, 0.53) | 0.07 (-0.38, 0.53) | 0.09 (-0.37, 0.54) | 0.07 (-0.38, 0.53) |
| Fitting model by two-piecewise linear regression |  |  |  |  |  |  |
| Inflection point of telomere length (per 0.1 change) | 7.33 | 7.97 | 7.97 | 7.98 | 7.98 | 7.98 |
| ≤ inflection point | 3.11 (-2.21, 8.43) | 1.98 (-0.98, 4.94) | 2.07 (-0.89, 5.04) | 2.04 (-0.91, 4.99) | 2.02 (-0.93, 4.97) | 2.04 (-0.90, 4.99) |
| > inflection point | 0.22 (-0.32, 0.76) | 0.05 (-0.46, 0.56) | 0.05 (-0.45, 0.56) | 0.03 (-0.48, 0.54) | 0.03 (-0.47, 0.54) | 0.04 (-0.47, 0.55) |
| P for log likelihood ratio test | 0.297 | 0.226 | 0.206 | 0.206 | 0.210 | 0.206 |

All models adjusted the same covariates, including age (Smooth); any CAD (self report); any family with heart attack or angina; sex; poverty to income ratio(Smooth); martial status; race; education; ALT (Smooth); AST (Smooth); blood urea nirogen (Smooth); creatinine (Smooth); Uric acid (Smooth); BMI (Smooth); physical activity; current or past cigarette smoker?; alcohol (Smooth); caffeine (Smooth); calcium (Smooth); carbohydrate (Smooth); cholesterol (Smooth); dietary fiber (Smooth); energy (Smooth); total monounsaturated fatty acids (Smooth); total polyunsaturated fatty acids (Smooth); total saturated fatty acids (Smooth); total fat (Smooth); any diabetes; any hypertension.

Supplement table 8: nonlinearity among pre- and pro-imputation data (Triglycerides vs telomere length)

|  | Pre-imputation | Pro-imputation1 | Pro-imputation2 | Pro-imputation3 | Pro-imputation4 | Pro-imputation5 |
| --- | --- | --- | --- | --- | --- | --- |
| Fitting model by standard linear regression | -1.19 (-2.55, 0.18) | -1.03 (-2.11, 0.05) | -1.00 (-2.08, 0.09) | -1.02 (-2.10, 0.07) | -0.99 (-2.07, 0.09) | -1.00 (-2.08, 0.08) |
| Fitting model by two-piecewise linear regression |  |  |  |  |  |  |
| Inflection point of telomere length (per 0.1 change) | 7.21 | 7.21 | 7.21 | 7.21 | 7.21 | 7.21 |
| ≤ inflection point | 7.07 (-9.03, 23.17) | 11.28 (-1.83, 24.39) | 10.86 (-2.24, 23.97) | 11.41 (-1.70, 24.52) | 11.51 (-1.60, 24.62) | 11.63 (-1.47, 24.74) |
| > inflection point | -1.77 (-3.21, -0.32) | -1.94 (-3.14, -0.74) | -1.90 (-3.10, -0.70) | -1.94 (-3.14, -0.74) | -1.93 (-3.13, -0.73) | -1.91 (-3.11, -0.70) |
| P for log likelihood ratio test | 0.291 | 0.053 | 0.071 | 0.058 | 0.053 | 0.057 |

All models adjusted the same covariates, including age (Smooth); any CAD (self report); any family with heart attack or angina; sex; poverty to income ratio(Smooth); martial status; race; education; ALT (Smooth); AST (Smooth); blood urea nirogen (Smooth); creatinine (Smooth); Uric acid (Smooth); BMI (Smooth); physical activity; current or past cigarette smoker?; alcohol (Smooth); caffeine (Smooth); calcium (Smooth); carbohydrate (Smooth); cholesterol (Smooth); dietary fiber (Smooth); energy (Smooth); total monounsaturated fatty acids (Smooth); total polyunsaturated fatty acids (Smooth); total saturated fatty acids (Smooth); total fat (Smooth); any diabetes; any hypertension.

Supplement table 9: nonlinearity among pre- and pro-imputation data (HDL-C vs telomere length)

|  | Pre-imputation | Pro-imputation1 | Pro-imputation2 | Pro-imputation3 | Pro-imputation4 | Pro-imputation5 |
| --- | --- | --- | --- | --- | --- | --- |
| Fitting model by standard linear regression | 0.17 (0.04, 0.31) | 0.15 (0.04, 0.27) | 0.15 (0.03, 0.26) | 0.14 (0.02, 0.25) | 0.14 (0.02, 0.25) | 0.15 (0.03, 0.26) |
| Fitting model by two-piecewise linear regression |  |  |  |  |  |  |
| Inflection point of telomere length (per 0.1 change) | 12.52 | 12.45 | 12.45 | 12.44 | 12.45 | 12.45 |
| ≤ inflection point | 0.50 (0.27, 0.73) | 0.48 (0.28, 0.67) | 0.48 (0.29, 0.68) | 0.46 (0.26, 0.66) | 0.47 (0.27, 0.66) | 0.47 (0.27, 0.67) |
| > inflection point | -0.22 (-0.53, 0.09) | -0.12 (-0.39, 0.14) | -0.13 (-0.39, 0.14) | -0.11 (-0.38, 0.15) | -0.12 (-0.39, 0.14) | -0.12 (-0.39, 0.14) |
| P for log likelihood ratio test | 0.001 | 0.002 | 0.002 | 0.004 | 0.003 | 0.003 |

All models adjusted the same covariates, including age (Smooth); any CAD (self report); any family with heart attack or angina; sex; poverty to income ratio(Smooth); martial status; race; education; ALT (Smooth); AST (Smooth); blood urea nirogen (Smooth); creatinine (Smooth); Uric acid (Smooth); BMI (Smooth); physical activity; current or past cigarette smoker?; alcohol (Smooth); caffeine (Smooth); calcium (Smooth); carbohydrate (Smooth); cholesterol (Smooth); dietary fiber (Smooth); energy (Smooth); total monounsaturated fatty acids (Smooth); total polyunsaturated fatty acids (Smooth); total saturated fatty acids (Smooth); total fat (Smooth); any diabetes; any hypertension.

Supplement table 10: Results of subgroup analysis and interaction test (LDL)

| : LDL-cholesterol (mg/dL) | β | 95%CI Low | 95%CI High | P(interaction) |
| --- | --- | --- | --- | --- |
| Age (years) |  |  |  | 0.325 |
| 19-40 | -0.19 | -0.87 | 0.49 |  |
| 41-60 | 0,12 | -0.76 | 1.00 |  |
| 61-80 | 1.43 | -0.24 | 3.09 |  |
| >80 | -1.12 | -5.57 | 3.33 |  |
| Sex |  |  |  | 0.560 |
| male | 0.12 | -0.64 | 0.88 |  |
| female | 0.42 | -0.28 | 1.13 |  |
| Race/ethnicity |  |  |  | 0.048# |
| Mexican American | -0.64 | -2.91 | 1.63 |  |
| Non-Hispanic Black | 2.43 | 0.80 | 4.05 |  |
| Non-Hispanic White | 0.25 | -0.35 | 0.85 |  |
| Other Hispanic | -1.07 | -3.23 | 1.09 |  |
| Other race/ethnicity | 0.33 | -4.49 | 5.15 |  |
| Education |  |  |  | 0.901 |
| less than high schoo | 0.29 | -1.13 | 1.71 |  |
| high school | 0.11 | -0.90 | 1.13 |  |
| more than high school | 0.39 | -0.29 | 1.06 |  |
| ALT (U/L) Tertile |  |  |  | 0.533 |
| Low | 0.48 | -0.44 | 1.40 |  |
| Middle | 0.52 | -0.34 | 1.39 |  |
| High | -0.11 | -1.01 | 0.79 |  |
| AST (U/L) Tertile |  |  |  | 0.492 |
| Low | 0.61 | -0.22 | 1.44 |  |
| Middle | 0.03 | -0.85 | 0.92 |  |
| High | -0.07 | -1.09 | 0.94 |  |
| Blood urea nitrogen (mg/dL) Tertile |  |  |  | 0.403 |
| Low | 0.08 | -0.98 | 1.15 |  |
| Middle | -0.05 | -0.88 | 0.77 |  |
| High | 0.74 | -0.18 | 1.66 |  |
| hyperuricemia |  |  |  | 0.377 |
| No | 0.42 | -0.14 | 0.99 |  |
| Yes | -0.22 | -1.54 | 1.11 |  |
| Alcohol intake |  |  |  | 0.505 |
| No | 0.17 | -0.43 | 0.76 |  |
| Yes | 0.57 | -0.46 | 1.60 |  |
| Caffeine (mg) Tertile |  |  |  | 0.817 |
| Low | -0.30 | -0.66 | 1.27 |  |
| Middle | 0.65 | -0.31 | 1.60 |  |
| High | 0.27 | -0.59 | 1.12 |  |
| Calcium (mg) Tertile |  |  |  | 0.731 |
| Low | 0.13 | -0.84 | 1.10 |  |
| Middle | 0.59 | -0.32 | 1.49 |  |
| High | 0.17 | -0.69 | 1.02 |  |
| Carbohydrate (gm) Tertile |  |  |  | 0.139 |
| Low | 0.01 | -1.02 | 1.04 |  |
| Middle | -0.34 | -1.20 | 0.51 |  |
| High | 0.84 | -0.03 | 1.71 |  |
| Cholesterol (mg) Tertile |  |  |  | 0.409 |
| Low | -0.16 | -1.04 | 0.72 |  |
| Middle | 0.29 | -0.59 | 1.17 |  |
| High | 0.70 | -0.26 | 1.67 |  |
| Dietary fiber (gm) Tertile |  |  |  | 0.946 |
| Low | 0.12 | -0.83 | 1.08 |  |
| Middle | 0.24 | -0.62 | 1.11 |  |
| High | 0.34 | -0.57 | 1.26 |  |
| Energy (kcal) Tertile |  |  |  | 0.143 |
| Low | 0.18 | -0.84 | 1.21 |  |
| Middle | -0.50 | -1.39 | 0.38 |  |
| High | 1.02 | 0.18 | 1.87 |  |
| Total monounsaturated fatty acids (gm) Tertile |  |  |  | 0.914 |
| Low | 0.27 | -0.78 | 1.31 |  |
| Middle | 0.09 | -0.86 | 1.04 |  |
| High | 0.35 | 0.44 | 1.14 |  |
| Total polyunsaturated fatty acids (gm) Tertile |  |  |  | 0.493 |
| Low | -0.09 | -1.08 | 0.89 |  |
| Middle | 0.10 | -0.77 | 0.98 |  |
| High | 0.64 | -0.22 | 1.50 |  |
| Total saturated fatty acids (gm) Tertile |  |  |  | 0.114 |
| Low | 0.85 | -0.18 | 1.89 |  |
| Middle | 0.99 | -1.91 | -0.06 |  |
| High | 0.87 | 0.06 | 1.67 |  |
| Total fat (gm) Tertile |  |  |  | 0.548 |
| Low | -0.07 | -1.09 | 0.95 |  |
| Middle | 0.25 | -0.71 | 1.21 |  |
| High | 0.62 | -0.18 | 1.42 |  |
| Creatinine (mg/dL) Tertile |  |  |  | 0.803 |
| Low | 0.61 | -0.54 | 1.76 |  |
| Middle | 0.16 | -0.63 | 0.94 |  |
| High | 0.34 | -0.54 | 1.23 |  |
| Body Mass Index (kg/m**2) |  |  |  | 0.3320 |
| underweight | -1.00 | -5.26 | 3.26 |  |
| normal | -0.23 | -0.99 | 0.54 |  |
| overweight | 0.35 | -0.49 | 1.18 |  |
| obesity | 0.81 | -0.15 | 1.77 |  |
| Physical Activity (MET-based rank) |  |  |  | 0.361 |
| Sits | -0.81 | -2.27 | 0.64 |  |
| Walks | 0.65 | -0.29 | 1.60 |  |
| Light loads | -0.12 | -1.43 | 1.18 |  |
| Heavy work | 0.27 | -0.56 | 1.11 |  |
| Any Diabetes |  |  |  | 0.299 |
| No | 0.18 | -0.34 | 0.71 |  |
| Yes | 1.63 | -1.09 | 4.35 |  |
| Any Hypertension |  |  |  | <0.001# |
| No | -0.27 | -0.83 | 0.29 |  |
| Yes | 2.53 | 1.41 | 3.65 |  |
| Any CAD |  |  |  | 0.991 |
| No | 0.22 | -0.29 | 0.74 |  |
| Yes | 0.26 | -6.52 | 7.04 |  |
| Martial Status NEW |  |  |  | 0.808 |
| married | 0.46 | -0.23 | 1.14 |  |
| single | 0.11 | -0.74 | 0.97 |  |
| living with partner | 0.08 | -2.45 | 2.61 |  |
| Any family with heart attack or angina |  |  |  | 0.584 |
| No | 0.24 | -0.43 | 0.90 |  |
| Yes | -0.05 | -0.86 | 0.76 |  |
| Current or Past Cigarette Smoker |  |  |  | 0.121 |
| none | 0.46 | -0.24 | 1.16 |  |
| current | 0.36 | -0.74 | 1.46 |  |
| past | -0.84 | -1.93 | 0.26 |  |
| Poverty to income ratio Tertile |  |  |  | 0.471 |
| Low | -0.21 | -1.28 | 0.87 |  |
| Middle | 0.64 | -0.32 | 1.60 |  |
| High | 0.08 | -0.70 | 0.85 |  |
| CRP |  |  |  | 0.669 |
| Low | 0.03 | -0.90 | 0.96 |  |
| Middle | 0.50 | -0.54 | 1.53 |  |
| High | 0.59 | -0.40 | 1.58 |  |

Dependent variable: LDL-C

BMI categories: underweight (<18.5kg/m^2^), normal (18.5-24.9kg/m^2^), overweight (25.0-29.9kg/m^2^), obesity (≥30kg/^m2^)

Above models adjusted for all covariates presented table 1.

In each case, the model is not adjusted for the stratification variable

Supplement table 11: Results of Subgroup analysis and interaction test (HDL)

| HDL-Cholesterol (mg/dL) | β. | 95%CI Low | 95%CI High | | P(interaction) |  |
| --- | --- | --- | --- | --- | --- | --- |
| Age (years) |  |  |  | | 0.222 |  |
| 19-40 | 0.04 | -0.16 | 0.24 | |  |  |
| 41-60 | 0.25 | 0.02 | 0.49 | |  |  |
| 61-80 | 0.20 | -0.24 | 0.64 | |  |  |
| >80 | -0.67 | -1.70 | 0.36 | |  |  |
| Sex |  |  |  | | 0.334 |  |
| male | 0.26 | 0.05 | 0.47 | |  |  |
| female | 0.12 | -0.07 | 0.31 | |  |  |
| RACE |  |  |  | | 0.046# |  |
| Mexican American | -0.14 | -0.75 | 0.47 | |  |  |
| Non-Hispanic Black | -0.04 | -0.48 | 0.40 | |  |  |
| Non-Hispanic White | 0.16 | -0.01 | 0.33 | |  |  |
| Other Hispanic | 0.93 | 0.38 | 1.48 | |  |  |
| Other race/ethnicity | 0.12 | -0.81 | 1.06 | |  |  |
| Education |  |  |  | | 0.153 |  |
| less than high schoo | 0.42 | 0.06 | 0.78 | |  |  |
| high school | -0.01 | -0.30 | 0.27 | |  |  |
| more than high school | 0.23 | 0.04 | 0.42 | |  |  |
| ALT (U/L) Tertile |  |  |  | | 0.087 |  |
| Low | -0.04 | -0.30 | 0.21 | |  |  |
| Middle | 0.19 | -0.04 | 0.42 | |  |  |
| High | 0.38 | 0.16 | 0.61 | |  |  |
| AST (U/L) Tertile |  |  |  | | 0.443 |  |
| Low | 0.21 | -0.02 | 0.44 | |  |  |
| Middle | 0.03 | -0.20 | 0.26 | |  |  |
| High | 0.21 | -0.10 | 0.43 | |  |  |
| Blood urea nitrogen (mg/dL) Tertile |  |  |  | | 0.118 |  |
| Low | 0.38 | 0.09 | 0.68 | |  |  |
| Middle | 0.01 | -0.22 | 0.25 | |  |  |
| High | 0.26 | 0.02 | 0.49 | |  |  |
| hyperuricemia |  |  |  | | 0.170 |  |
| No | 0.22 | 0.06 | 0.38 | |  |  |
| Yes | -0.06 | -0.42 | 0.31 | |  |  |
| Alcohol intake |  |  |  | | 0.534 |  |
| No | 0.16 | -0.01 | 0.32 | |  |  |
| Yes | 0.25 | -0.01 | 0.52 | |  |  |
| Caffeine (mg) Tertile |  |  |  | | 0.353 |  |
| Low | 0.22 | -0.03 | 0.48 | |  |  |
| Middle | 0.32 | 0.07 | 0.58 | |  |  |
| High | 0.07 | -0.17 | 0.31 | |  |  |
| Calcium (mg) Tertile |  |  |  | | 0.08 |  |
| Low | 0.25 | -0.02 | 0.51 | |  |  |
| Middle | 0.40 | 0.15 | 0.65 | |  |  |
| High | 0.01 | -0.22 | 0.25 | |  |  |
| Carbohydrate (gm) Tertile |  |  |  | | 0.512 |  |
| Low | 0.24 | -0.03 | 0.51 | |  |  |
| Middle | 0.30 | 0.06 | 0.54 | |  |  |
| High | 0.11 | -0.13 | 0.34 | |  |  |
| Cholesterol (mg) Tertile |  |  |  | | 0.106 |  |
| Low | 0.47 | 0.22 | 0.73 | |  |  |
| Middle | 0.06 | -0.19 | 0.31 | |  |  |
| High | 0.09 | -0.16 | 0.34 | |  |  |
| Dietary fiber (gm) Tertile |  |  |  | | 0.682 |  |
| Low | 0.23 | -0.02 | 0.48 | |  |  |
| Middle | 0.23 | -0.01 | 0.47 | |  |  |
| High | 0.09 | -0.16 | 0.35 | |  |  |
| Energy (kcal) Tertile |  |  |  | | 0.275 |  |
| Low | 0.18 | -0.09 | 0.45 | |  |  |
| Middle | 0.39 | 0.14 | 0.64 | |  |  |
| High | 0.12 | -0.11 | 0.35 | |  |  |
| Total monounsaturated fatty acids (gm) Tertile |  |  |  | | 0.285 |  |
| Low | 0.39 | 0.11 | 0.66 | |  |  |
| Middle | 0.11 | 0.14 | 0.36 | |  |  |
| High | 0.14 | -0.09 | 0.36 | |  |  |
| Total polyunsaturated fatty acids (gm) Tertile |  |  |  | | 0.221 |  |
| Low | 0.39 | 0.13 | 0.66 | |  |  |
| Middle | 0.08 | -0.16 | 0.33 | |  |  |
| High | 0.17 | -0.07 | 0.41 | |  |  |
| Total saturated fatty acids (gm) Tertile |  |  |  | | 0.300 |  |
| Low | 0.42 | 0.14 | 0.70 | |  |  |
| Middle | 0.10 | -0.08 | 0.41 | |  |  |
| High | 0.17 | -0.06 | 0.40 | |  |  |
| Total fat (gm) Tertile |  |  |  | | 0.100 |  |
| Low | 0.44 | 0.17 | 0.71 | |  |  |
| Middle | 0.20 | -0.06 | 0.45 | |  |  |
| High | 0.06 | -0.16 | 0.29 | |  |  |
| Creatinine (mg/dL) Tertile |  |  |  | | 0.326 |  |
| Low | 0.39 | 0.08 | 0.69 | |  |  |
| Middle | 0.11 | -0.11 | 0.32 | |  |  |
| High | 0.21 | -0.23 | 0.21 | |  |  |
| Body Mass Index (kg/m^2^) |  |  |  | | 0.546 |  |
| underweight | -0.72 | -2.30 | 0.86 | |  |  |
| normal | 0.13 | -0.10 | 0.37 | |  |  |
| overweight | 0.27 | 0.01 | 0.52 | |  |  |
| obesity | 0.25 | -0.01 | 0.52 | |  |  |
| Physical Activity (MET-based rank) |  |  |  | | 0.343 |  |
| Sits | 0.11 | -0.24 | 0.47 | |  |  |
| Walks | 0.31 | 0.04 | 0.57 | |  |  |
| Light loads | 0.40 | 0.06 | 0.74 | |  |  |
| Heavy work | 0.07 | -0.16 | 0.31 | |  |  |
| Any Diabetes |  |  |  | | 0.965 |  |
| No | 0.22 | 0.07 | 0.36 | |  |  |
| Yes | 0.20 | -0.43 | 0.83 | |  |  |
| Any Hypertension |  |  |  | | 0.346 |  |
| No | 0.24 | 0.08 | 0.40 | |  |  |
| Yes | 0.07 | -0.24 | 0.38 | |  |  |
| Any CAD |  |  |  | | 0.962 |  |
| No | 0.21 | 0.06 | 0.35 | |  |  |
| Yes | 0.18 | -1.07 | 1.43 | |  |  |
| Martial Status |  |  |  | | 0.147 |  |
| married | 0.32 | 0.13 | 0.51 | |  |  |
| single | 0.09 | -0.15 | 0.33 | |  |  |
| living with partner | -0.21 | -0.87 | 0.45 | |  |  |
| Any family with heart attack or angina |  |  |  | | 0.461 |  |
| No | 0.25 | 0.07 | 0.43 | |  |  |
| Yes | 0.14 | -0.09 | 0.37 | |  |  |
| Current or Past Cigarette Smoker |  |  |  | | 0.016# |  |
| none | 0.05 | -0.14 | 0.24 | |  |  |
| current | 0.42 | 0.11 | 0.73 | |  |  |
| past | 0.50 | 0.20 | 0.380 | |  |  |
| Poverty to income ratio Tertile |  |  |  | | 0.333 |  |
| Low | 0.37 | 0.09 | 0.65 | |  |  |
| Middle | 0.08 | -0.18 | 0.34 | |  |  |
| High | 0.22 | 0.00 | 0.43 | |  |  |
| CRP |  |  | |  | 0.304 | |
| Low | 0.16 | -0..08 | | 0.40 |  | |
| Middle | -0.11 | -0.37 | | 0.15 |  | |
| High | 0.03 | -0.22 | | 0.28 |  | |

Dependent variable: HDL-C

BMI categories: underweight (<18.5kg/m^2^), normal (18.5-24.9kg/m^2^), overweight (25.0-29.9kg/m^2^), obesity (≥30kg/^m2^)

Above models adjusted for all covariates presented table 1.

In each case, the model is not adjusted for the stratification variable

Supplement table 12: Results of subgroup analysis and interaction test

| Triglycerides (mg/dL) | β | 95%CI Low | 95%CI High | P(interaction) |
| --- | --- | --- | --- | --- |
| Age (years) |  |  |  | 0.136 |
| 19-40 | -0.34 | -2.21 | 1.54 |  |
| 41-60 | -3.18 | -5.40 | -0.96 |  |
| 61-80 | -0.26 | -4.32 | 3.81 |  |
| >80 | 0.02 | -8.99 | 9.03 |  |
| Sex |  |  |  | 0.911 |
| male | -1.24 | -3.28 | 0.80 |  |
| female | -1.09 | -2.93 | 0.76 |  |
| Race/ethnicity |  |  |  | 0.158 |
| Mexican American | -1.40 | -7.13 | 4.34 |  |
| Non-Hispanic Black | -0.31 | -4.42 | 3.80 |  |
| Non-Hispanic White | -0.75 | -2.34 | 0.84 |  |
| Other Hispanic | -6.59 | -11.75 | -1.44 |  |
| Other race/ethnicity | 4.75 | -4.07 | 13.57 |  |
| Education |  |  |  | 0.552 |
| less than high schoo | -1.40 | -4.82 | 2.02 |  |
| high school | 0.01 | -2.68 | 2.70 |  |
| more than high school | -1.76 | -3.56 | 0.04 |  |
| ALT (U/L) Tertile |  |  |  | 0.096 |
| Low | -1.29 | -3.84 | 1.26 |  |
| Middle | -0.95 | -3.24 | 1.33 |  |
| High | -1.38 | -3.62 | 0.86 |  |
| AST (U/L) Tertile |  |  |  | 0.118 |
| Low | -3.16 | -5.49 | 0.83 |  |
| Middle | 0.12 | -2.21 | 2.46 |  |
| High | -0.58 | -3.02 | 1.87 |  |
| Blood urea nitrogen (mg/dL) Tertile |  |  |  | 0.129 |
| Low | -0.49 | -3.32 | 2.35 |  |
| Middle | 0.01 | -2.21 | 2.23 |  |
| High | -3.06 | -5.30 | -0.81 |  |
| hyperuricemia |  |  |  | 0.073 |
| No | -1.73 | -3.17 | -0.22 |  |
| Yes | 1.44 | -1.76 | 4.63 |  |
| Alcohol intake |  |  |  | 0.109 |
| No | -1.81 | -3.43 | -0.20 |  |
| Yes | 0.62 | -1.92 | 3.16 |  |
| Caffeine (mg) Tertile |  |  |  | 0.489 |
| Low | -0.04 | -2.47 | 2.38 |  |
| Middle | -1.72 | -4.16 | 0.73 |  |
| High | -1.89 | -4.14 | 0.36 |  |
| Calcium (mg) Tertile |  |  |  | 0.179 |
| Low | -0.53 | -3.07 | 2.01 |  |
| Middle | -2.91 | -5.24 | -0.58 |  |
| High | -0.05 | -2.26 | 2.17 |  |
| Carbohydrate (gm) Tertile |  |  |  | 0.222 |
| Low | -2.93 | -5.52 | -0.34 |  |
| Middle | -1.23 | -3.52 | 1.06 |  |
| High | 0.08 | -2.18 | 2.34 |  |
| Cholesterol (mg) Tertile |  |  |  | 0.442 |
| Low | -2.45 | -4.71 | -0.19 |  |
| Middle | -2.14 | -1.09 | 3.37 |  |
| High | -2.31 | -4.57 | -0.05 |  |
| Dietary fiber (gm) Tertile |  |  |  | 0.918 |
| Low | -1.25 | -3.62 | 1.11 |  |
| Middle | -0.99 | -3.31 | 1.32 |  |
| High | -0.56 | -2.97 | 1.86 |  |
| Energy (kcal) Tertile |  |  |  | 0.968 |
| Low | -1.11 | -3.65 | 1.44 |  |
| Middle | -1.54 | -3.94 | 0.86 |  |
| High | -1.25 | -3.42 | 0.93 |  |
| Total monounsaturated fatty acids (gm) Tertile |  |  |  | 0.083 |
| Low | -2.41 | -5.07 | 0.26 |  |
| Middle | 0.92 | -1.48 | 3.32 |  |
| High | -2.34 | -4.48 | -0.20 |  |
| Total polyunsaturated fatty acids (gm) Tertile |  |  |  | 0.661 |
| Low | -0.68 | -3.20 | 1.84 |  |
| Middle | -2.10 | -4.41 | 0.22 |  |
| High | -0.89 | -3.11 | 1.33 |  |
| Total saturated fatty acids (gm) Tertile |  |  |  | 0.752 |
| Low | -2.05 | -4.70 | 0.60 |  |
| Middle | -0.70 | -3.04 | 1.64 |  |
| High | -1.35 | -3.53 | 0.84 |  |
| Total fat (gm) Tertile |  |  |  | 0.220 |
| Low | -2.61 | -5.17 | -0.06 |  |
| Middle | 0.41 | -2.00 | 2.82 |  |
| High | -1.49 | -3.64 | 0.66 |  |
| Creatinine (mg/dL) Tertile |  |  |  | 0.900 |
| Low | -1.63 | -4.74 | 1.48 |  |
| Middle | -1.47 | -3.62 | 0.67 |  |
| High | -0.89 | -3.03 | 1.24 |  |
| Body Mass Index (kg/m^2^) |  |  |  | 0.146 |
| underweight | 2.88 | -12.04 | 17.79 |  |
| normal | -1.74 | -3.96 | 0.48 |  |
| overweight | -2.68 | -5.07 | -0.29 |  |
| obesity | 1.09 | -1.40 | 3.59 |  |
| Physical Activity (MET-based rank) |  |  |  | 0.526 |
| Sits | -2.48 | -5.80 | 0.84 |  |
| Walks | 0.12 | -2.41 | 2.65 |  |
| Light loads | -2.37 | -5.55 | 0.80 |  |
| Heavy work | -1.51 | -3.73 | 0.71 |  |
| Any Diabetes |  |  |  | 0.292 |
| No | -0.95 | -2.33 | 0.42 |  |
| Yes | -4.22 | -10.16 | 1.72 |  |
| Any Hypertension |  |  |  | 0.172 |
| No | -1.52 | -3.04 | 0.01 |  |
| Yes | 0.80 | -2.17 | 3.77 |  |
| Any CAD |  |  |  | 0.918 |
| No | -1.28 | -2.66 | 0.09 |  |
| Yes | -0.66 | -12.53 | 11.21 |  |
| Martial Status |  |  |  | 0.454 |
| married | -0.93 | -2.70 | 0.83 |  |
| single | -1.60 | -3.87 | 0.68 |  |
| living with partner | 2.59 | -3.64 | 8.81 |  |
| Any family with heart attack or angina |  |  |  | 0.464 |
| No | -4.02 | -5.32 | -2.72 |  |
| Yes | 0.38 | -1.75 | 2.51 |  |
| Current or Past Cigarette Smoker |  |  |  | 0.001# |
| none | 0.27 | -1.53 | 2.07 |  |
| current | -6.08 | -9.02 | -3.13 |  |
| past | -1.02 | -3.80 | 1.76 |  |
| Poverty to income ratio Tertile |  |  |  | 0.721 |
| Low | -2.13 | -4.80 | 0.54 |  |
| Middle | -0.94 | -3.44 | 1.56 |  |
| High | -0.82 | -2.87 | 1.23 |  |
| CRP |  |  |  | 0.605 |
| Low | -1.92 | -4.09 | 0.25 |  |
| Middle | -0.43 | -2.82 | 1.97 |  |
| High | -0.66 | -2.97 | 1.65 |  |

Dependent variable: TG

BMI categories: underweight (<18.5kg/m^2^), normal (18.5-24.9kg/m^2^), overweight (25.0-29.9kg/m^2^), obesity (≥30kg/^m2^)

Above models adjusted for all covariates presented table 1.

In each case, the model is not adjusted for the stratification variable
